# Effects of copper in *Daphnia* are modulated by nanosized titanium dioxide and natural organic matter: what is the impact of aging duration?

**DOI:** 10.1007/s11356-020-11578-2

**Published:** 2020-11-17

**Authors:** Rajdeep Roy, Simon Lüderwald, Asawer Alawi Ahmed Maknoon, George Metreveli, Ralf Schulz, Mirco Bundschuh

**Affiliations:** 1 iES Landau, Institute for Environmental Sciences, University of Koblenz-Landau, Fortstrasse 7, 76829 Landau, Germany; 2 Department of Aquatic Sciences and Assessment, Swedish University of Agricultural Sciences, Uppsala, Sweden

**Keywords:** Aging, Nanomaterials, Natural organic matter, Metal ions, Combined effect, Mixture toxicity

## Abstract

During its aquatic life cycle, nanosized titanium dioxide (nTiO_2_) may interact with natural organic matter (NOM) ultimately altering the ecotoxicity of co-occurring chemical stressors such as heavy metals (e.g. copper (Cu)). In this context, the following hypotheses were tested: (1) aging of nTiO_2_ along with Cu reduces Cu toxicity, (2) nTiO_2_ agglomerates have a lower potential to reduce Cu toxicity and (3) aging of nTiO_2_ in presence of NOM reduces Cu toxicity further. A multifactorial test design crossing three nTiO_2_ levels (0.0, 0.6 and 3.0 mg/L) with two levels of NOM (0 versus 8 mg total organic carbon (TOC)/L) and seven nominal Cu concentrations (ranging from 0 to 1536 μg/L) aged in ASTM medium for 0, 1, 3 and 6 days was realised, while two aging scenarios were applied (type 1: nTiO_2_ jointly aged with Cu; type 2: Cu added after nTiO_2_ aging). Subsequently, Cu toxicity was assessed using the immobility of *Daphnia magna* after 48 h of exposure as response variable. The experiments revealed that neither aging duration nor the extent of agglomeration (type 1 vs. type 2 aging) has a substantial impact on Cu induced toxicity. Moreover, it was confirmed that the presence of NOM substantially reduced Cu induced toxicity, independent of the aging scenario and duration. More importantly, the data suggest the ingestion of Cu loaded nTiO_2_ as additional exposure pathway contributing to Cu toxicity. In conclusion, it seems unlikely that nTiO_2_ concentrations currently detected in or predicted for aquatic ecosystems, which are at least one order of magnitude below the concentration tested here, influence Cu toxicity meaningfully.

**Supplementary Information:**

The online version contains supplementary material available at 10.1007/s11356-020-11578-2.

## Introduction

As a consequence of their small size, nanoparticles (NPs) possess physical and chemical features that are fundamentally different from their water-soluble or bulk counterparts. Those features include a high specific surface area, reactivity in liquid or gas phase and rapid diffusion (Nowack and Bucheli 2007; Fan et al. 2011) making nanotechnology a multitrillion dollars business involving approximately 6 million employees worldwide (He et al. 2019). Furthermore, the Nanotechnology Consumer Products Inventory (CPI) listed 1814 consumer products from 6222 companies of 32 countries in 2015 (Mohajerani et al. 2019). This success goes along with an estimated annual production of NPs of approximately 300,000 metric tons in 2010. From this amount, 21%, 17% and 2.5% are assumed to be released at different stages of the nanoparticle’s life cycle into water, soils and air, respectively (Keller et al. 2013; Keller and Lazareva 2014). In the environment, NPs interact with a range of natural (such as natural organic matter (NOM)) and anthropogenic substances including dissolved heavy metals that may represent a hazard for the integrity of freshwater ecosystems (Millennium Ecosystem Assessment 2005) raising concerns about their joint environmental health risk.

Indeed, some publications have reported increased metal toxicity in the presence of NPs (e.g. Fan et al. 2011; Wang et al. 2011; Rosenfeldt et al. 2014), while others highlight a reduction (Chen et al. 2015; Canesi et al. 2015). This remediation like effect may be explained by the adsorption of heavy metal ions onto the NPs surface, followed by an intraparticle diffusion, ultimately reducing heavy metal toxicity (Qu et al. 2013). The discrepancy of NP-induced changes in metal toxicity may depend on the NP concentration (Wang et al. 2011) as well as the metal identity or more specifically the metal ion’s charge (Rosenfeldt et al. 2014). It was, moreover, highlighted that the combined toxicity of metals and NP depends on the environmental conditions triggering their fate and interaction over time (Rosenfeldt et al. 2016). Indeed, Rosenfeldt et al. (2016) highlighted that Cu toxicity was reduced with increasing aging duration in presence of titanium dioxide (nTiO_2_) relative to NP’s absence. While this pattern was observed in medium with ionic strength (9.25 mmol/L), distilled water (ionic strength ~ 0 mmol/L) did not influence Cu toxicity. The authors also documented that the presence of NOM could contribute to mitigation of Cu toxicity through complexation, an effect that is elevated in presence of nTiO_2_. At the same time, Seitz et al. (2015) documented that the presence of NOM during nTiO_2_ aging can slightly and transiently increase NP toxicity. These studies jointly suggest a complex interplay of NOM, NP and metal ions over time warranting further research targeting the temporal dynamics of this interaction and ultimately their consequence for aquatic biota.

**Fig. 1:**
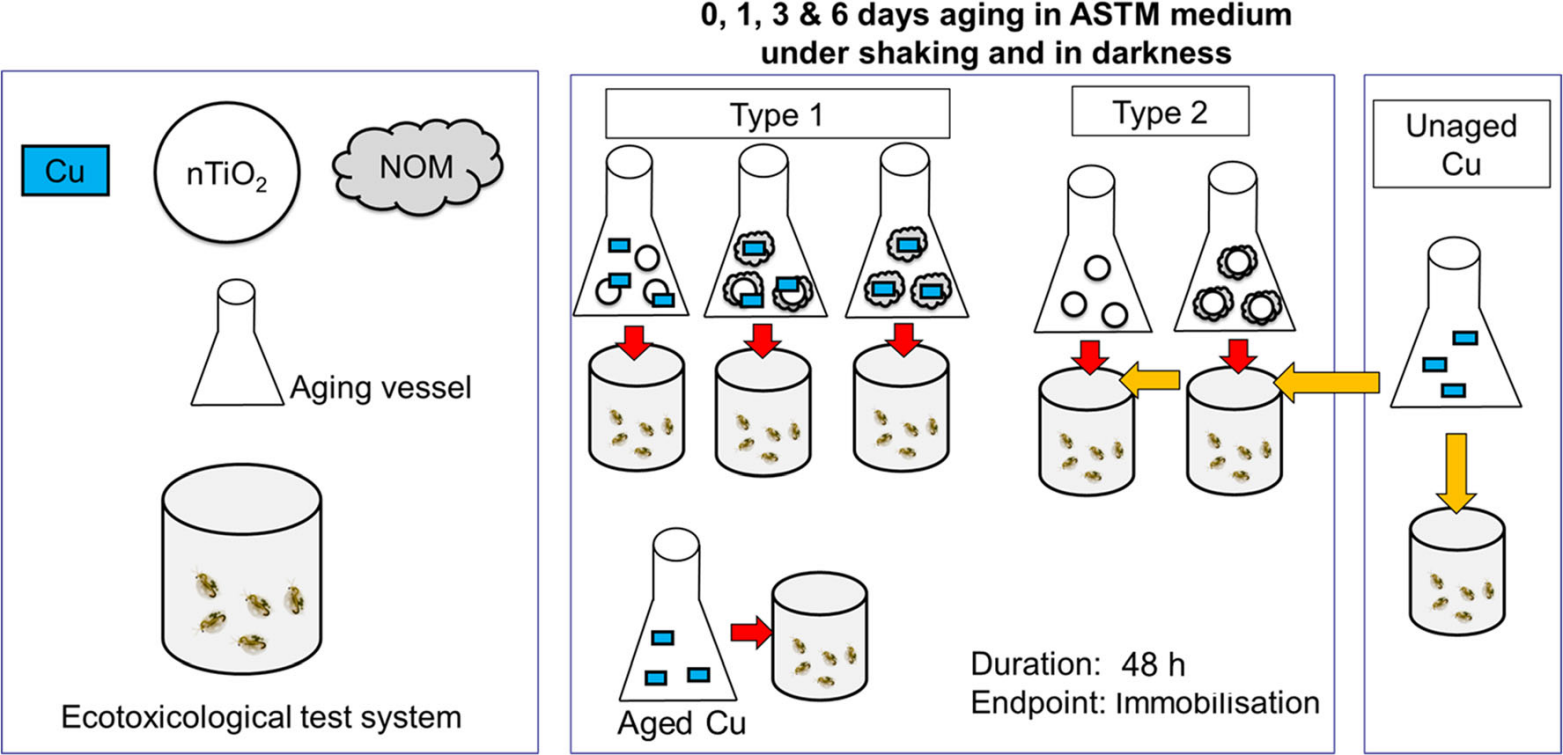
Schematic diagram visualizing the two aging scenarios (type 1 and type 2) assessing the impact of variable aging durations (i.e. 0, 1, 3 and 6 days) of nTiO_2_ in combination with NOM on the toxicity of co-aged or freshly added Cu towards *D. magna*. The test assessing for the sensitivity baseline of *D. magna* is also visualised (unaged Cu)

In the present study, the consequences of nTiO_2_ aging (0, 1, 3 and 6 days) at three concentrations (i.e. reflecting the 96 h EC_10_ and EC_50_ of nTiO_2_ (Fig. 1S) for *Daphnia* as well as the absence of nTiO_2_) in the presence and absence of NOM (seaweed extract as used in many earlier papers, namely Rosenfeldt et al. 2015a, 2016) on Cu toxicity (seven concentrations from the range 0–1536 μg/L; using a spacing factor of 2) were assessed for the model species *Daphnia magna*. Thereby, two aging scenarios in ASTM reconstituted hard water (ASTM 2007) were realised prior to the toxicity assessment: the first scenario ensured the interaction of nTiO_2_ with Cu in presence and absence of NOM during aging. The second scenario focused on the aging of nTiO_2_ and NOM (presence vs. absence) with Cu being spiked to the test medium just before the introduction of the test species. These aging scenarios simulate the interaction of Cu with fresh nTiO_2_ (often in their nanoform) or with aged and thus agglomerated particles, whose average particle size is often in the μm-range (Cupi et al. 2015; Seitz et al. 2015; Rosenfeldt et al. 2016), allowing to assess for the importance of the particle or agglomerate size. The selection of nTiO_2_ as model NP is motivated by its high production volume (Jovanović 2015) and widespread application (Baiqi et al. 2006; Chen and Mao 2007; Mueller and Nowack 2008). This NP has additionally only limited acute toxicity for *Daphnia*, which is even further reduced by NOM (Seitz et al. 2015). Copper (Cu) served as model heavy metal interfering with the sodium (Na^+^) regulation and metabolism in freshwater animals (Grosell and Wood 2002; Grosell et al. 2002), whose toxicity can also be reduced in presence of NOM (Al-Reasi et al. 2012) and potentially nTiO_2_ (Liu et al. 2015). Additionally, Cu has a broad application range, including agriculture leading to surface water levels in the high micrograms per litre range (Süß et al. 2006).

In the present study, it was hypothesised that (1) aging of nTiO_2_ along with Cu reduces Cu toxicity with increasing aging duration as a consequence of a reduced Cu bioavailability at test initiation (Rashidi et al. 2010). It was, moreover, assumed that (2) the aging of nTiO_2_ in the absence of Cu limits the NPs possibility to reduce effects of Cu when freshly added to the test medium just before toxicity testing. This is likely triggered by a lower surface area of NP agglomerates and the short interaction time before the test organisms experience Cu exposure (Aydın et al. 2008). Finally, it was presumed that (3) aging of nTiO_2_ in the presence of NOM reduces Cu toxicity with increasing aging duration. This pattern is triggered by NOM colloidally stabilising nTiO_2_ extending its interaction time with Cu, ultimately reducing Cu effects (Lee et al. 2011). Moreover, NOM forms complexes with metal ions contributing to reduced Cu toxicity (Rosenfeldt et al. 2015a).

## Materials and methods

### Chemicals

A stable nTiO_2_ dispersion, produced by stirred media milling (PML 2; Bühler AG, Switzerland) of P25 nTiO_2_ powder (AEROXIDE® TiO_2_ P25; advertised primary particle size of 21 nm and surface area of 50 ± 15 m^2^/g; Evonik) in deionised water, was provided by the Institute for Particle Technology (TU Braunschweig, Germany). The stock dispersion was stabilised at low pH (~3), exhibiting a monodisperse size distribution and an average particle diameter of approximately 80 nm (Electronic supplementary material, Table 1S). The nTiO_2_ stock dispersion (2000 mg/L) was ultra-sonicated for 10 min prior use, to ensure a homogeneous particle distribution. The dissolved Cu stock solution was prepared separately for each experimental run using Cu(NO_3_)_2_ × 3H_2_O (Carl Roth; purity ≥ 99%, p.a., ACS). The salt was mixed with deionised water in a volumetric flask (polypropylene), followed by serial dilution to obtain the seven nominal test Cu concentrations ranging from 0 to 1536 μg/L (Tables 2S and 3S). The exact concentration range depended on the aging scenario and was selected to obtain a full dose-response curve. Dissolved Cu species including Cu^2+^, CuOH^+^ and Cu_2_(OH)_2_^2+^ (Rosenfeldt et al. 2016) are hereafter summarised as Cu.

### Test species

The test species *D. magna* (clone V, Eurofins-GAB laboratories, Germany) was cultured in ASTM reconstituted hard water modified with selenium and vitamins (biotin, thiamine, cyanocobalamin) according to OECD 202 (OECD 2004) and 8 mg TOC/L seaweed extract (Marinure®, Glenside, Scotland) (ASTM 2007). The culture medium was changed three times per week, and the organisms were fed with the green algae *Desmodesmus* sp. at an equivalent of 200 μg C per organism and day. The culture was maintained in a climate-controlled chamber (Weiss Environmental Technology Inc., Germany) at 20 ± 1 °C and a 16:8 h light:dark rhythm (800–1000 lx; OSRAM L 58 W/21–840 ECO, Germany). At the initiation of the bioassays, newly hatched juveniles (age < 24 h) were collected and randomly introduced into the respective replicates using pasture pipette.

### Experimental setup

The impact of nTiO_2_ on the toxicity of Cu was assessed under two aging scenarios involving a 3-factorial (2 × 3 × 7) experimental design each (Fig. 1). The aging procedures were performed due to space limitations at 16 ± 1 °C and thus at a lower temperature relative to the toxicity bioassays (as the behaviour of nTiO_2_ was not impacted at the lower temperature (Table 4S), this was considered acceptable), in darkness on a horizontal shaker (50 rpm; VKS-B-50, Edmund Bühler GmbH, Germany). The conditions during aging should prevent photo-activation of nTiO_2_ and consequently the oxidation of NOM (Seitz et al. 2015). Moreover, the constant shaking avoided sedimentation of nTiO_2_ and thereby ensured its constant availability in the water phase to interact with NOM and Cu. In contrast to Rosenfeldt et al. (2016), who aged Cu, nTiO_2_ and NOM as stock solutions, we performed aging in test medium (ASTM) (Table 5S) at nutrient, Cu, nTiO_2_ and NOM concentrations as applied during toxicity testing, which avoided any impact of changes in the environmental conditions on the fate of Cu or nTiO_2_. During the first scenario (type 1), nTiO_2_ was aged at three levels (0.0, 0.6 and 3.0 mg/L) along with Cu (seven concentrations from the range 0–1536 μg/L; using a spacing factor of 2) in combination with NOM (0 vs. 8 mg TOC/L; Table 2S). After 0 (~ 15 min), 1, 3 and 6 days of aging (Rosenfeldt et al. 2016), the medium (or dispersion of nTiO_2_ in combination with Cu and NOM) was used for toxicity testing (see *bioassays*). The second scenario (type 2) aged nTiO_2_ at the same three concentrations in presence or absence of NOM (8 mg TOC/L) for 0 (~ 15 min), 1, 3 and 6 days. Subsequently, the aged medium was transferred to the respective replicates of the bioassay, followed by addition of the respective Cu concentration (Table 3S) and test organisms. Irrespective of the aging scenario, the aged medium was homogenised by stirring at 350 rpm for 2 min before separation into replicates. Additionally, the mean hydrodynamic diameter of the NPs was determined (Table 6S) at the bioassay initiation using dynamic light scattering (DelsaNano C, Beckman Coulter, Germany).

The concentrations of Cu were expected to change during aging, with its magnitude depending on the concentration of nTiO_2_ and NOM (Fan et al. 2016). Consequently, Cu levels were analytically determined after aging at the start of the bioassays for the first aging scenario (type 1). As Cu was spiked to the aged medium just before the bioassay initiation for all experiments of the second scenario (type 2), accurate Cu spiking was anticipated based on the analytical results of the first aging scenario following 0 days of aging. Similarly, we quantified nTiO_2_ concentrations (Table 7S) only for one of the exposure scenarios (0 day aged samples) assuming a similar deviation between nominal and measured concentrations for other factor combinations. Quantification of nTiO_2_ and Cu at a later stage of the bioassays was not realised as the present study indicated that relevant interactions between nTiO_2_ and Cu occurred during the first hours (see Table 8S, “Results” and “Discussion”).

The high number of acute toxicity tests required a temporal separation of the experiments, called experimental runs. To allow for a direct comparison of *Daphnia* responses among experimental runs, an additional dose-response experiment was performed during each experimental run quantifying the sensitivity of *D. magna* to unaged Cu (without nTiO_2_ and NOM). The EC_50_ values obtained from these experiments were ultimately used as a sensitivity baseline allowing the quantification of changes in Cu toxicity depending on the aging conditions.

### Bioassays

Per replicate, five juvenile daphnids (<24 h age) were introduced into the aged ASTM medium containing respective concentrations of nTiO_2_, Cu and NOM and exposed for a duration of up to 96 h. The immobilisation of *Daphnia* was recorded every 24 h (i.e. 24 h, 48 h, 72 h and 96 h). The acute toxicity tests were performed in polystyrene beakers (filled with 50 mL of aged test medium) under controlled laboratory conditions (20 ± 1 °C with a 16:8 h light:dark rhythm) with four independent replicates per treatment, largely following OECD 202 (OECD 2004).

### Quantification of nTiO_2_ and Cu

The concentrations of nTiO_2_ or Cu in the aged medium were quantified according to the method described by Rosenfeldt et al. (2015b) with minor modification. Briefly, after aging (1, 3 and 6 days), 10 mL supernatant was taken from the water phase after centrifugation (4000 rpm for 10 min) of the aged medium. Centrifugation forced nTiO_2_ to sediment, ensuring the separation of Cu from NPs. The concentration of Cu adsorbed on the surface of nTiO_2_ was not analytically confirmed. After that, the samples were acidified with HNO_3_ (65%) and stored at 4 °C. However, in the case of 0 day aging, samples with nTiO_2_ were acidified with HCl (35%) to avoid agglomeration and analysed without centrifugation. The samples were analysed by Inductively Coupled Plasma Optical Emission Spectrometry (ICP-OES; Agilent 720, Germany) using the wavelengths of 336.12 and 327.39 nm for Ti and Cu quantification, respectively. The limit of detection was 2 μg/L for both Ti and Cu. The measured concentration of nTiO_2_ (recalculated based on Ti quantification) deviated from their nominal levels by less than 20% justifying the use of the latter throughout the study (see Table 7S).

### Statistical analysis

**Fig. 2:**
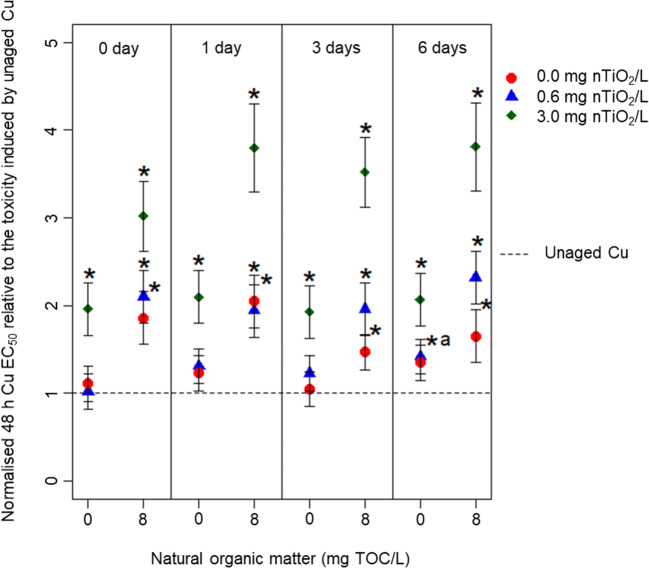
Changes in the Cu 48 h EC_50_ of *D. magna* normalised to the toxicity induced by the unaged Cu solution (the reference absolute 48 h EC_50_ range 54.6 ± 9.1 to 66.7 ± 13.1 μg/L) and reflect the impact of nTiO_2_ (0.0, 0.6 or 3.0 mg/L), NOM (0 or 8 mg/L) and aging duration (0, 1, 3, 6 days) for the type 1 aging scenario. The EC_50_ values are based on nominal Cu concentrations. Asterisk indicates a significant difference of the respective EC_50_ value relative to the bioassays testing for the effects of unaged Cu. The ‘a’ indicates a significant difference of the respective EC_50_ values relative to the same combinations of treatments but aged for 0 days

**Fig. 3:**
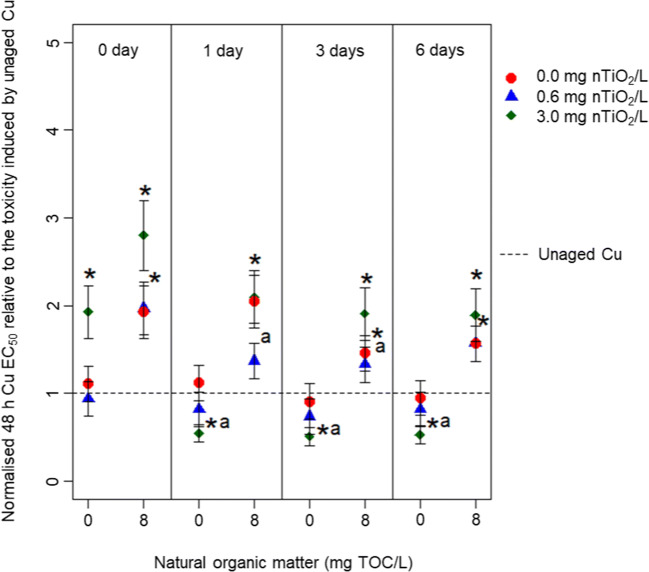
Changes in the Cu 48 h EC_50_ of *D. magna* normalised to the toxicity induced by the unaged Cu solution (the reference absolute 48 h EC_50_ range 47.9 ± 8.0 to 58.6 ± 11.5 μg/L) and reflect the impact of nTiO_2_ (0.0, 0.6 or 3.0 mg/L), NOM (0 or 8 mg/L) and aging duration (0, 1, 3, 6 days) for the type 1 aging scenario. The EC_50_ values are based on measured Cu concentrations. Asterisk indicates a significant difference of the respective EC_50_ value relative to the bioassays testing for the effects of unaged Cu. The ‘a’ indicates a significant difference of the respective EC_50_ values relative to the same combinations of treatments but aged for 0 days

**Fig. 4:**
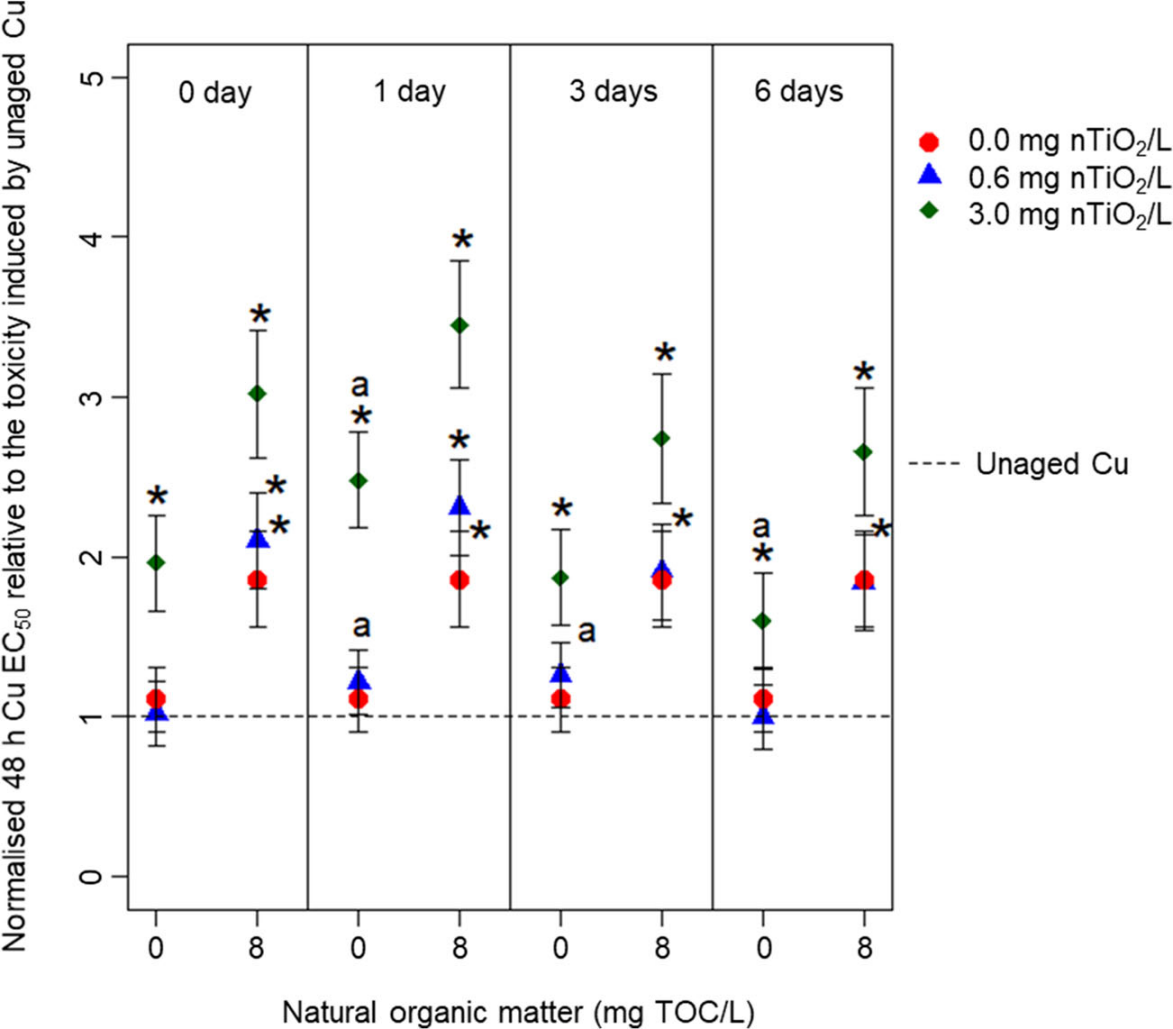
Changes in the Cu 48 h EC_50_ of *D. magna* normalised to the toxicity induced by the unaged Cu solution (the reference absolute 48 h EC_50_ range 54.6 ± 9.1 to 59.7 ± 2.0 μg/L) and reflect the impact of nTiO_2_ (0.0, 0.6 or 3.0 mg/L), NOM (0 or 8 mg/L) and aging duration (0, 1, 3, 6 days) for the type 2 aging scenario. The EC_50_ values are based on nominal Cu concentrations. Asterisk indicates a significant difference of the respective EC_50_ value relative to the bioassays testing for the effects of unaged Cu. The ‘a’ indicates a significant difference of the respective EC_50_ values relative to the same combinations of treatments but aged for 0 days

The statistical analyses of this study were performed with R version 3.0.1 (for windows) and the extension package drc (Ritz and Streibig 2005; R Core Team 2013). EC_50_ values, the half median concentration causing immobilisation of 50% of daphnids, together with their 95% confidence interval were abstracted from dose-response models, while the model fitting the data best was selected by means of Akaike’s information criterion (Tables 9S and 10S) and expert judgement. Model building was in a first-place based on nominal Cu concentrations (Figs. 2S–8S; Table 9S) and subsequently normalised to the effective (= measured) Cu concentration quantified in the water phase at bioassay initiation (Figs. 9S–15S; Tables 8S and 10S). The latter step should aid data interpretation as to inform about negative effects on *Daphnia*, which may not be directly explainable by Cu water phase concentrations. All comparisons discussed in this paper are based on 48 h Cu EC_50_ values, as effects caused by Cu after 24 h, 72 h and 96 h of exposure were either low (24 h) or already disguised by nTiO_2_ toxicity (72 h and 96 h) (Dabrunz et al. 2011). Models and model parameters are reported in the electronic supplementary material (Figs. 2S–15S; Tables 9S and 10S). The EC_50_ values of each aging situation were evaluated for statistically significant differences as compared with the respective EC_50_ value of unaged Cu solution, as well as relative to the respective EC_50_ value of unaged (0 day aging) situation using 95% confidence interval testing (Wheeler et al. 2006). If 95% CIs of the difference between two EC_50_ values did not include zero, the difference was considered statistically significant (Rosenfeldt et al. 2014).

## Results

### Type 1 aging scenario

Based on nominal Cu concentrations (Fig. 2), 3.0 mg nTiO_2_/L reduced Cu toxicity approx. 2-fold, relative to its absence, whereas 0.6 mg nTiO_2_/L did not change Cu toxicity after 0, 1, 3 and 6 days of aging. The presence of NOM significantly increased EC_50_ values independent of aging duration up to 2-fold in the absence of nTiO_2_ relative to the unaged Cu treatment.

Adjusting the Cu EC_50_ values to the Cu concentration, confirmed analytically in the water phase (Fig. 3), uncovered that in the absence of NOM at 0.0 and 0.6 mg nTiO_2_/L, hardly any alteration occurred relative to the unaged Cu treatment. Contrary, the presence of 3.0 mg nTiO_2_/L initially (0 days of aging) led to a 2-fold reduction, but after 1, 3 and 6 days of aging, it turned into a 2-fold increase in Cu toxicity. The amendment of NOM to the aging medium caused a reduction in Cu toxicity by 1.5–2-fold at 0.0 and 0.6 mg nTiO_2_/L, which was independent of the aging duration. In the presence of 3.0 mg nTiO_2_/L in combination with 8 mg NOM/L, in contrast, a reduction in Cu toxicity was observed reaching a factor of up to three. This mitigation like effect continuously decreased to a factor of two, approaching 48 h EC_50_ values comparable with 6 days of aging at 0.0 and 0.6 mg nTiO_2_/L.

### Type 2 aging scenario

Aging of nTiO_2_ with or without NOM before adding Cu and the test organisms (Fig. 4; see based on measured concentration in Fig. 16S) resulted in a similar pattern as discussed for the nominal concentrations when applying the type 1 aging scenario (Fig. 2). In general, while NOM reduced Cu toxicity, the low nTiO_2_ concentration had no additional mitigating effect neither in presence nor in the absence of NOM. Only at 3.0 mg nTiO_2_/L, a further reduction in Cu toxicity was observed. These patterns were independent of the aging duration.

## Discussion

### Influence of nTiO_2_ on Cu toxicity in the absence of NOM

The changes in Cu-induced toxicity in presences of nTiO_2_ is likely triggered by the NP’s negative surface charge (approx. − 9 mV in absence of NOM as documented by (Rosenfeldt et al. 2015a) attracting positively charged Cu species (e.g. Cu^2+^, CuOH^+^, Cu_2_(OH)_2_^2+^) (Rosenfeldt et al. 2016). Although the present study does not address the underlying chemical mechanisms of the observations reported here, it clearly displays that the concentration of nTiO_2_ is an important driver influencing Cu toxicity. At 0.6 mg nTiO_2_/L, there was hardly any difference in Cu toxicity relative to the NP’s absence. The presence of 3.0 mg nTiO_2_/L, in contrast, caused substantial changes in Cu-induced toxicity. Besides the NP concentration, also the particle size, an indicator for the available surface area, might trigger the magnitude of changes in Cu toxicity (Rosenfeldt et al. 2015a). As the particle size was comparable at the start as well as at the termination of the aging procedure among the two applied NP concentrations (Table 6S), the latter determines the surface area available for interactions with Cu and thus not the particle size. Consequently, the lower nTiO_2_ concentration provided insufficient surface area to reduce Cu concentrations in the water phase (Table 8S) and, thus, its effects in *Daphnia*. The type 2 aging scenario supports this interpretation, namely that the concentration of nTiO_2_ is the main driver in the present study for the observed effects: We aged nTiO_2_ in the absence of Cu, which increased its particle size (Table 6S), before interacting with Cu. The mitigation potential of nTiO_2_ for Cu-induced toxicity did not substantially change with increasing aging duration and thus particles size, while the observed effects were comparable among aging scenarios (Figs. 2 and 4). Moreover, the interaction between Cu and nTiO_2_ seems to be a rapid process highlighted by the reduction in Cu toxicity independent of the aging scenario and aging duration. However, as the Cu concentration was only quantified at the start of the *Daphnia* bioassay but not during the actual bioassay, this requires further evaluation. Conjointly, the first and second hypotheses suggesting an impact of aging time and agglomeration size of nTiO_2_ for Cu mitigation need to be rejected.

As indicated above, the direction of Cu toxicity changed (i.e. increased) when based on its analytically verified concentration at the start of the experiment with *D. magna*, relative to its nominal concentration in the presence of 3.0 mg nTiO_2_/L under the type 1 aging scenario. As the nTiO_2_ concentration tested here did not induce any mortality after comparable exposure durations when tested individually (Fig. 1S), these differences point to additional exposure pathways relative to the waterborne exposure alone. In other words, if the Cu water phase concentration would explain the observed toxicity, the Cu EC_50_ value should be comparable with those obtained in the absence of NOM and nTiO_2_ and without aging (baseline Cu toxicity experiments). This pattern would have remained unnoticed when the assessment is based exclusively on Cu concentrations confirmed before aging—in fact, the Cu concentration measured in the water phase would lead to an underestimation of acute effects. As additional exposure pathway, *Daphnia* may have ingested NPs together with Cu. During gut passage (at pH = neutral), Cu may have been remobilised as a consequence of enzyme (including protease, amylase, lipase and cellulase) activity (Weltens et al. 2000; Rosenfeldt et al. 2014). Ultimately, this pathway interacts with the waterborne Cu exposure explaining the increased toxicity when based on measured concentrations (Fig. 3).

### Influence of NOM on Cu toxicity

The reduction in Cu induced toxicity in the presence of NOM and independent of the aging scenario or duration can mainly be explained by the formation of Cu chelates. These Cu chelates are formed through the interaction with carboxylic and phenolic groups of NOM, ultimately reducing the bioavailability of Cu (Lorenzo et al. 2002; Rosenfeldt et al. 2015a; Yu et al. 2018). In the presence of nTiO_2_, NOM further decreased the toxicity triggered by Cu (Figs. 2 and 4). This pattern, which is in line with our third hypothesis, may be driven by a coating of the NP’s surface (Fig. 17S) with hydrophobic or aromatic components of the NOM (Lee et al. 2011; Pakarinen et al. 2013). This process is, on the one hand, known to reduce agglomeration and thus stabilises nTiO_2_ (see Table 6S) through electrostatic repulsion in the water phase (Loosli et al. 2014). Thereby, the available surface area for Cu adsorption is increased relative to the absence of NOM. Moreover, the presence of hydrophobic or aromatic organic material on nTiO_2_ surfaces further enhances the surface charge (from approx. − 9 mV in absence to − 20 mV in presence of NOM, respectively, as documented by Rosenfeldt et al. 2015a), which may have stimulated Cu adsorption and complexation. Those processes, however, in combination with the direct interaction of Cu and NOM in the water phase, might have contributed to the observed decrease in Cu toxicity. This assumption is also supported when comparing the EC_50_ values based on measured and nominal Cu concentrations during the type 1 aging scenario (Figs. 2 and 3). The fact that the EC_50_ values—particularly after aging in the presence of the high nTiO_2_ concentration— are substantially reduced when based on measured relative to nominal concentrations (Figs. 2 and 3) points towards the significant Cu adsorption onto NOM coated nTiO_2_. Furthermore, the EC_50_ values based on measured Cu concentrations in the presence of NOM were higher relative to the absence of both NOM and nTiO_2_. This outcome suggests that Cu complexation is reducing its toxicity by roughly 50% (Fig. 3).

Nonetheless, the uptake of Cu complexed by NOM on nTiO_2_ surfaces is possible in *Daphnia* considering their particle size distribution (Table 6S) exceeds the mesh size (240–640 nm) of *Daphnia*’s filtering apparatus (Seitz et al. 2015). However, the Cu on the NP’s surface seems to be less bioavailable in presence relative to the absence of NOM (Figs. 2 and 3) as the EC_50_ value based on measured Cu concentrations indicated a reduced toxicity in the presence of NOM relative to the elevated toxicity in the absence of NOM.

In conclusion, from the present study, it is evident that field relevant concentrations of nTiO_2_, namely far below the lowest concentration of 0.6 mg/L as tested here, are unlikely to influence Cu toxicity significantly. Despite a trend to an increased Cu toxicity is observed in the presence of 0.6 mg nTiO_2_/L but absence of NOM, this assumption seems reasonable particularly as in surface water bodies, NOM is ubiquitous, which triggered a reduction in Cu toxicity in our study. Moreover, we can see that the aging duration does not meaningfully affect the direction and magnitude of Cu induced effects pointing to a rapid interaction between NOM, Cu and nTiO_2_. This rapid interaction also suggests that changes in Cu toxicity in the long run, are rather unlikely— at least under stable environmental conditions as tested here. Nonetheless, the present study highlights the possibility of NPs to increase toxicity if Cu is taken up during gut passage, a pathway of exposure largely overlooked in the assessment of combined effects of particulate stressors (nanoparticles or nanoplastics) and dissolved chemical stressors. Additionally, the direct effects of nTiO_2_ seems of little relevance for the actual toxicity assessment as the toxicity of the NPs tested in the present study only appeared after 96 h of exposure. Finally, it is suggested to assess for the transferability of the results generated here to other heavy metals (with variable ionic charge) to allow for a general interpretation of the relevance of this process.

## Supplementary information

ESM 1 (PDF 1.41 mb).

## Data Availability

All data generated or analysed during this study are included in this published research article and its electronic supplementary material.
