# Supplementary material for: Effects of copper in Daphnia are modulated by nanosized titanium dioxide and natural organic matter: what is the impact of aging duration?
Source: Environ Sci Pollut Res Int. 2020 Nov 17;28(11):13991–9. doi: 10.1007/s11356-020-11578-2 (PMC7943511; doi:10.1007/s11356-020-11578-2)
Supplement: Supplementary file 1 — (PDF 1.41 mb). [file 11356_2020_11578_MOESM1_ESM.pdf]

Electronic supplementary material

Environmental Science and Pollution Research

Effects of copper in *Daphnia* are modulated by nanosized titanium dioxide and natural organic matter: what is the impact of aging duration?

Rajdeep Roy* [1], Simon Lüderwald [1], Asawer Alawi Ahmed Maknoon [1], George Metreveli [1], Ralf Schulz [1] and Mirco Bundschuh* [1, 2]

[1] iES Landau, Institute for Environmental Sciences, University of Koblenz-Landau, Landau, Germany

[2] Department of Aquatic Sciences and Assessment, Swedish University of Agricultural Sciences, Uppsala, Sweden

*Corresponding authors:

Rajdeep Roy & Mirco Bundschuh

iES Landau, Institute for Environmental Sciences

University of Koblenz-Landau

Fortstrasse 7, 76829 Landau, Germany

Email: rajdeep@uni-landau.de; bundschuh@uni-landau.de

Table 1S Particle size distribution, i.e. average hydrodynamic diameter (nm, mean ± SE) polydispersity index (PI); 10th, 50th and 90th percentile (D10%, D50%, D90%), of P25 $nTiO_2$ (2000 mg/L) measured in the stock suspension (n = 3)

Size (nm)	PI	10th percentile	50th percentile	90th percentile
79.1 ± 0.8	0.20 ± 0.00	39.5 ± 3.8	85.1 ± 1.1	191.4 ± 20.7

Table 2S The nominal concentrations of the test elements for the treatments of the type 1 aging scenario

Aging duration (days)	$nTiO_2$ (mg/L)	NOM (mg TOC/L)	Cu concentrations (μg/L)
0	0.6	0	0, 6, 12, 24, 48, 96, 192
		8	0, 24, 48, 96, 192, 384, 768
	3.0	0	0, 6, 12, 24, 48, 96, 192
		8	0, 48, 96, 192, 384, 768, 1536
	0.0	0	0, 12, 24, 48, 96, 192, 384
		8	0, 24, 48, 96, 192, 384, 768
1	0.6	0	0, 12, 24, 48, 96, 192, 384
		8	0, 24, 48, 96, 192, 384, 768
	3.0	0	0, 12, 24, 48, 96, 192, 384
		8	0, 24, 48, 96, 192, 384, 768
	0.0	0	0, 12, 24, 48, 96, 192, 384
		8	0, 24, 48, 96, 192, 384, 768
3	0.6	0	0, 24, 48, 96, 192, 384, 768
		8	0, 48, 96, 192, 384, 768, 1536
	3.0	0	0, 24, 48, 96, 192, 384, 768
		8	0, 48, 96, 192, 384, 768, 1536
	0.0	0	0, 12, 24, 48, 96, 192, 384
		8	0, 24, 48, 96, 192, 384, 768
6	0.6	0	0, 24, 48, 96, 192, 384, 768
		8	0, 48, 96, 192, 384, 768, 1536
	3.0	0	0, 24, 48, 96, 192, 384, 768
		8	0, 48, 96, 192, 384, 768, 1536
	0.0	0	0, 12, 24, 48, 96, 192, 384
		8	0, 24, 48, 96, 192, 384, 768

Table 3S The nominal concentrations of the test elements for the treatments of the type 2 aging scenario

Aging duration (days)	nTiO2 (mg/L)	NOM (mg TOC/L)	Cu concentrations (µg/L)
0	0.6	0	0, 6, 12, 24, 48, 96, 192
		8	0, 24, 48, 96, 192, 384, 768
	3.0	0	0, 6, 12, 24, 48, 96, 192
		8	0, 48, 96, 192, 384, 768, 1536
	0.0	0	0, 12, 24, 48, 96, 192, 384
		8	0, 24, 48, 96, 192, 384, 768
1	0.6	0	0, 12, 24, 48, 96, 192, 384
		8	0, 24, 48, 96, 192, 384, 768
	3.0	0	0, 12, 24, 48, 96, 192, 384
		8	0, 24, 48, 96, 192, 384, 768
3	0.6	0	0, 24, 48, 96, 192, 384, 768
		8	0, 48, 96, 192, 384, 768, 1536
	3.0	0	0, 24, 48, 96, 192, 384, 768
		8	0, 48, 96, 192, 384, 768, 1536
6	0.6	0	0, 24, 48, 96, 192, 384, 768
		8	0, 48, 96, 192, 384, 768, 1536
	3.0	0	0, 24, 48, 96, 192, 384, 768
		8	0, 48, 96, 192, 384, 768, 1536

Table 4S Preliminary assessment on hydrodynamic diameter (mean ± SE) of $nTiO_2$ (0.6 mg/L) in aging medium (ASTM) after few hours of aging process at 16 °C and 20 °C temperatures (n = 3)

Temperature (°C)	Duration (Hours)	NOM (mg TOC /L)	Cu (µg/L)	Diameter (nm)	PI
16	0	0	0	2612.5 ± 78.8	0.99 ± 0.02
			60	2692.8 ± 110.1	0.97 ± 0.04
		8	0	967.2 ± 37.2	0.40 ± 0.02
	24	0	0	2397.9 ± 237.9	0.80 ± 0.05
			60	2556.2 ± 58.9	0.88 ± 0.02
		8	0	959.9 ± 28.6	0.41 ± 0.00
	48	0	0	2433.4 ± 232.9	0.83 ± 0.05
			60	2622.5 ± 240.4	0.89 ± 0.05
		8	0	783.7 ± 34.5	0.35 ± 0.01
20	0	0	0	2672.1 ± 227.4	0.87 ± 0.05
			60	2730.8 ± 37.5	0.95 ± 0.03
		8	0	974.2 ± 9.9	0.41 ± 0.00
	24	0	0	2653.3 ± 57.7	0.94 ± 0.02
			60	2404.4 ± 142.0	0.87 ± 0.02
		8	0	862.6 ± 54.3	0.38 ± 0.02
	48	0	0	2267.5 ± 81.2	0.83 ± 0.03
			60	2673.6 ± 119.5	0.98 ± 0.01
		8	0	804.2 ± 39.8	0.36 ± 0.01

Table 5S Chemical composition, pH and ionic strength of ASTM medium (Seitz et al. 2015)

Ingredient	Concentration (mg/L)
$NaHCO_3$	192
$CaSO4 \times 2H_2O$	120
$MgSO_4$	120
KCl	8
Na_2SeO_3	0.00219
Thiamine hydrochloride (B_1)	0.075
Biotin (B_7)	0.00075
Cyanocobalamin (B_{12})	0.001
pH	~8.2
Ionic strength	9.25 mmol/L

Table 6S Hydrodynamic diameter (mean ± SE) of $nTiO_2$ in aging medium (ASTM) after the aging process (n = 3)

$nTiO_2$ (mg/L)	Aging duration (days)	NOM (mg TOC /L)	Cu (µg/L)	Diameter (nm)	PI
0.6	0	0	0	666.5 ± 41.8	0.29 ± 0.01
			192	724.6 ± 71.4	0.30 ± 0.02
		8	0	462.3 ± 0.7	0.21 ± 0.00
			768	422.6 ± 16.4	0.20 ± 0.00
	1	0	0	2349.9 ± 188.7	0.72 ± 0.04
			384	3051.4 ± 129.9	0.91 ± 0.02
		8	0	1595.7 ± 52.6	0.58 ± 0.01
			768	1556.4 ± 203.5	0.54 ± 0.05
	3	0	0	3095.7 ± 77.3	0.88 ± 0.04
			384	3501.3 ± 269.2	0.98 ± 0.03
		8	0	1312.2 ± 61.5	0.50 ± 0.01
			768	1614.6 ± 203.9	0.59 ± 0.06
	6	0	0	3381.5 ± 386.5	0.90 ± 0.07
			384	4237.1 ± 654.6	1.18 ± 0.14
		8	0	1503.0 ± 86.1	0.58 ± 0.02
			768	1068.1 ± 37.6	0.42 ± 0.01
3.0	0	0	0	1153.0 ± 340.8	0.39 ± 0.09
			192	1488.3 ± 168.2	0.50 ± 0.04
		8	0	279.4 ± 6.2	0.25 ± 0.01
			1536	290.2 ± 3.4	0.25 ± 0.00
	1	0	0	1904.0 ± 145.2	0.57 ± 0.04
			384	2024.1 ± 95.9	0.61 ± 0.02
		8	0	1011.8 ± 25.2	0.37 ± 0.01
			768	716.0 ± 41.8	0.28 ± 0.01
	3	0	0	2495.6 ± 96.4	0.71 ± 0.01
			384	2838.1 ± 203.1	0.76 ± 0.04
		8	0	1090.3 ± 40.4	0.40 ± 0.01

		768	723.7 ± 15.6	0.28 ± 0.00
6	0	0	3551.5 ± 333.4	0.85 ± 0.01
		384	3922.4 ± 547.9	0.93 ± 0.09
	8	0	1085.2 ± 96.7	0.42 ± 0.02
		768	891.2 ± 25.7	0.33 ± 0.00

Table 7S Measured concentration (mean ± SE) of $nTiO_2$ in the aging medium (n = 3)

Nominal concentration (mg/L)	NOM (mg TOC/L)	Measured concentration (mg/L)
0.6	0	0.7 ± 0.001
	8	0.6 ± 0.01
3.0	0	3.4 ± 0.01
	8	3.2 ± 0.01

Table 8S The measured (μg/L) and relative (% of the nominal, mean ± SE) concentration of Cu in the water phase of the aged dispersion or aged solution after the aging process (n = 3)

Aging duration (days)	$nTiO_2$ (mg/L)	NOM (mg TOC/L)	Cu concentration		
			Nominal (μg/L)	Measured (μg/L)	Relative (%)
0	0.6	0	48	38.61 ± 0.10	80.45 ± 0.22
		8	96	78.91 ± 0.25	82.20 ± 0.26
	3.0	0	96	83.24 ± 0.15	86.71 ± 0.16
		8	96	78.07 ± 0.16	81.32 ± 0.17
	0.0	0	48	42.14 ± 0.11	87.79 ± 0.24
		8	96	87.47 ± 1.13	91.11 ± 1.17
1	0.6	0	48	26.32 ± 0.19	54.84 ± 0.41
		8	96	59.40 ± 0.04	61.87 ± 0.04
	3.0	0	96	22.09 ± 0.08	23.01 ± 0.08
		8	192	93.26 ± 0.06	48.57 ± 0.03
	0.0	0	96	76.80 ± 0.05	80.00 ± 0.05
		8	96	84.06 ± 0.79	87.56 ± 0.82
3	0.6	0	48	25.43 ± 0.08	52.98 ± 0.17
		8	96	57.46 ± 0.01	59.86 ± 0.01
	3.0	0	96	22.36 ± 0.13	23.30 ± 0.14
		8	192	91.65 ± 0.08	47.73 ± 0.04
	0.0	0	96	72.97 ± 0.01	76.01 ± 0.01
		8	96	83.33 ± 2.21	86.80 ± 2.30
6	0.6	0	48	24.48 ± 0.03	51.00 ± 0.06
		8	96	56.89 ± 0.02	59.26 ± 0.02
	3.0	0	96	21.80 ± 0.04	22.71 ± 0.04
		8	192	83.70 ± 0.08	43.59 ± 0.04
	0.0	0	96	59.30 ± 0.53	61.77 ± 0.55
		8	96	80.29 ± 0.87	83.63 ± 0.91

Table 9S Model specification and respective Akaike's Information Criterion (AIC) for the calculation of 48 h Cu EC_{50} (based on nominal Cu concentrations)

Test items	Aging duration (day)	Aging scenario	Model	AIC
0.6 mg $nTiO_2$/L and Cu	0		Two-parameter Weibull function W2.2	– 25.68
	1	Type 1	Log-logistic dose-response model LL.2	– 106.01
	1	Type 2	Log-normal dose-response model LN.2	– 62.82
	3	Type 1	Log-normal dose-response model LN.2	– 40.61
	3	Type 2	Two-parameter Weibull function W2.2	– 62.62
	6	Type 1	Log-normal dose-response model LN.2	– 78.43
	6	Type 2	Log-normal dose-response model LN.2	– 78.56
0.6 mg $nTiO_2$/L, Cu and NOM	0		Two-parameter Weibull function W2.2	– 48.70
	1	Type 1	Log-normal dose-response model LN.2	– 106.01
	1	Type 2	Two-parameter Weibull function W1.2	– 78.82
	3	Type 1	Log-normal dose-response model LN.2	– 69.63
	3	Type 2	Log-normal dose-response model LN.2	– 69.64
	6	Type 1	Log-normal dose-response model LN.2	– 48.99
	6	Type 2	Log-logistic dose-response model LL.2	– 54.33

3.0 mg $nTiO_2$/L and Cu	0		Log-logistic dose-response model LL.2	– 60.94
	1	Type 1	Two-parameter Weibull function W2.2	– 46.44
	1	Type 2	Log-logistic dose-response model LL.2	– 72.36
	3	Type 1	Log-logistic dose-response model LL.2	– 69.71
	3	Type 2	Log-normal dose-response model LN.2	– 40.58
	6	Type 1	Log-normal dose-response model LN.2	– 86.61
	6	Type 2	Log-normal dose-response model LN.2	– 73.26
3.0 mg $nTiO_2$/L, Cu and NOM	0		Log-normal dose-response model LN.2	– 78.55
	1	Type 1	Log-normal dose-response model LN.2	– 59.15
	1	Type 2	Log-normal dose-response model LN.2	– 43.54
	3	Type 1	Log-normal dose-response model LN.2	– 62.89
	3	Type 2	Log-normal dose-response model LN.2	– 62.89
	6	Type 1	Log-normal dose-response model LN.2	– 48.98
	6	Type 2	Log-normal dose-response model LN.2	– 82.30
Unaged Cu	0		Log-normal dose-response model LN.2	– 69.64
	1	Type 1	Log-normal dose-response model LN.2	– 97.96
	1	Type 2	Two-parameter Weibull function W1.2	– 82.29

	3	Type 1	Two-parameter Weibull function W1.2	– 106.02
	3	Type 2	Log-normal dose-response model LN.2	– 106.01
	6	Type 1	Log-normal dose-response model LN.2	– 69.64
	6	Type 2	Log-normal dose-response model LN.2	– 97.96
0.0 mg $nTiO_2$/L and Cu	0		Log-normal dose-response model LN.2	– 70.08
0.0 mg $nTiO_2$/L, Cu and NOM	0		Log-normal dose-response model LN.2	– 78.55
0.0 mg $nTiO_2$/L and Cu	1	Type 1	Log-normal dose-response model LN.2	– 62.82
0.0 mg $nTiO_2$/L, Cu and NOM	1	Type 1	Log-normal dose-response model LN.2	– 81.77
0.0 mg $nTiO_2$/L and Cu	3	Type 1	Log-normal dose-response model LN.2	– 54.33
0.0 mg $nTiO_2$/L, Cu and NOM	3	Type 1	Log-normal dose-response model LN.2	– 106.01
0.0 mg $nTiO_2$/L and Cu	6	Type 1	Log-normal dose-response model LN.2	– 81.73
0.0 mg $nTiO_2$/L, Cu and NOM	6	Type 1	Two-parameter Weibull function W2.2	– 50.22

Table 10S Model specification and respective Akaike's Information Criterion (AIC) for the calculation of 48 h Cu EC_{50} (considering the measured Cu concentrations)

Test items	Aging duration (day)	Aging scenario	Model	AIC
0.6 mg $nTiO_2$/L and Cu	0		Two-parameter Weibull function W2.2	– 25.68
	1	Type 1	Log-logistic dose-response model LL.2	– 106.01
	1	Type 2	Log-normal dose-response model LN.2	– 62.82
	3	Type 1	Log-normal dose-response model LN.2	– 40.61
	3	Type 2	Two-parameter Weibull function W2.2	– 62.62
	6	Type 1	Log-normal dose-response model LN.2	– 78.43
	6	Type 2	Log-normal dose-response model LN.2	– 78.56
0.6 mg $nTiO_2$/L, Cu and NOM	0		Two-parameter Weibull function W2.2	– 48.70
	1	Type 1	Log-normal dose-response model LN.2	– 106.01
	1	Type 2	Two-parameter Weibull function W1.2	– 78.82
	3	Type 1	Log-normal dose-response model LN.2	– 69.63
	3	Type 2	Log-normal dose-response model LN.2	– 69.64
	6	Type 1	Log-normal dose-response model LN.2	– 48.99
	6	Type 2	Log-logistic dose-response model LL.2	– 54.33

3.0 mg $nTiO_2$/L and Cu	0		Log-logistic dose-response model LL.2	– 60.94
	1	Type 1	Two-parameter Weibull function W2.2	– 46.44
	1	Type 2	Log-logistic dose-response model LL.2	– 72.36
	3	Type 1	Log-logistic dose-response model LL.2	– 69.71
	3	Type 2	Log-normal dose-response model LN.2	– 40.58
	6	Type 1	Log-normal dose-response model LN.2	– 86.61
	6	Type 2	Log-normal dose-response model LN.2	– 73.26
3.0 mg $nTiO_2$/ L, Cu and NOM	0		Log-normal dose-response model LN.2	– 78.55
	1	Type 1	Log-normal dose-response model LN.2	– 59.15
	1	Type 2	Log-normal dose-response model LN.2	– 43.54
	3	Type 1	Log-normal dose-response model LN.2	– 62.89
	3	Type 2	Log-normal dose-response model LN.2	– 62.89
	6	Type 1	Log-normal dose-response model LN.2	– 48.98
	6	Type 2	Log-normal dose-response model LN.2	– 82.30
Unaged Cu	0		Log-normal dose-response model LN.2	– 69.64
	1	Type 1	Log-normal dose-response model LN.2	– 97.96
	1	Type 2	Two-parameter Weibull function W1.2	– 82.29

	3	Type 1	Two-parameter Weibull function W1.2	– 106.02
	3	Type 2	Log-normal dose-response model LN.2	– 106.01
	6	Type 1	Log-normal dose-response model LN.2	– 69.64
	6	Type 2	Log-normal dose-response model LN.2	– 97.96
0.0 mg $nTiO_2$/L and Cu	0		Log-normal dose-response model LN.2	– 70.08
0.0 mg $nTiO_2$/L, Cu and NOM	0		Log-normal dose-response model LN.2	– 78.55
0.0 mg $nTiO_2$/L and Cu	1	Type 1	Log-normal dose-response model LN.2	– 62.82
0.0 mg $nTiO_2$/L, Cu and NOM	1	Type 1	Log-normal dose-response model LN.2	– 81.77
0.0 mg $nTiO_2$/L and Cu	3	Type 1	Log-normal dose-response model LN.2	– 54.33
0.0 mg $nTiO_2$/L, Cu and NOM	3	Type 1	Log-normal dose-response model LN.2	– 106.01
0.0 mg $nTiO_2$/L and Cu	6	Type 1	Log-normal dose-response model LN.2	– 81.73
0.0 mg $nTiO_2$/L, Cu and NOM	6	Type 1	Two-parameter Weibull function W2.2	– 50.22

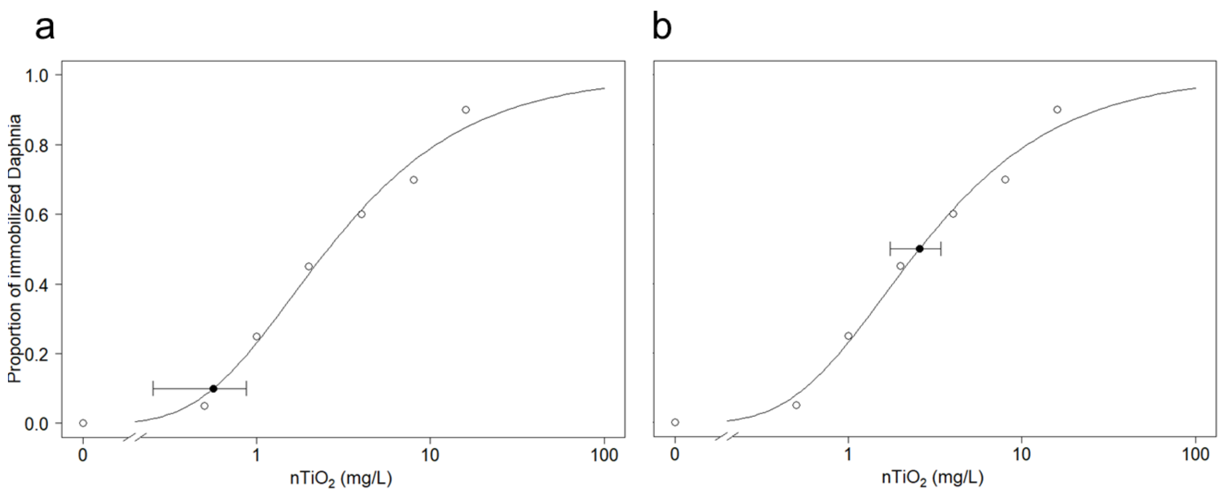

Fig. 1S Dose-Response curves of the 96 h (a) EC_{10} and (b) EC_{50} calculations for juvenile (< 24 h age) *D. magna* exposed to $nTiO_2$ concentrations (0, 0.5, 1, 2, 4, 8 and 16 mg/L). The mean mortality for each treatment is denoted by an open circle, while the filled circle indicates the (a) EC_{10} and (b) EC_{50} values of $nTiO_2$ along with 95% confidence interval. Additionally, no notable mortality was observed for *Daphnia* during 24 h, 48 h and 72 h assessments

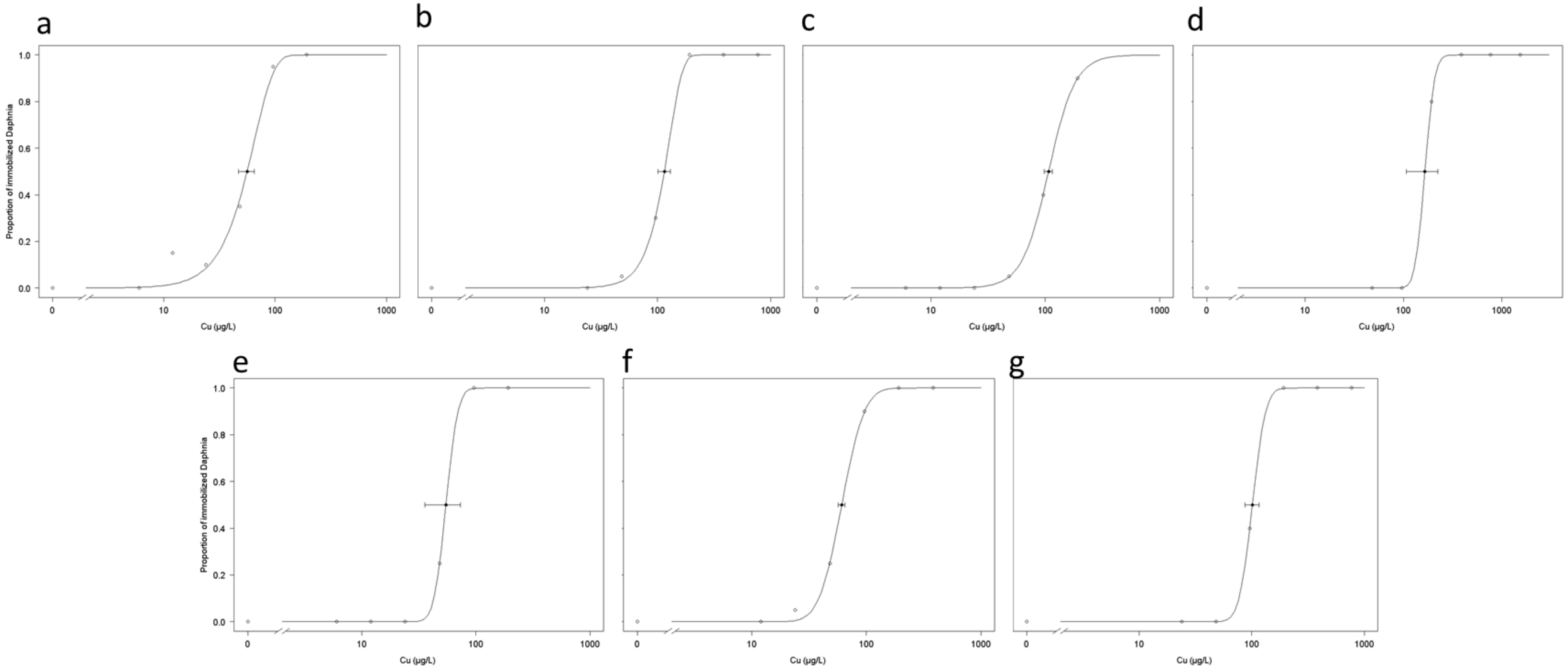

Fig. 2S Dose-Response curves based on nominal Cu concentrations for the 0 day aging in combination with (a) 0.6 mg $nTiO_2$/L, (b) 0.6 mg $nTiO_2$/L and NOM, (c) 3.0 mg $nTiO_2$/L, (d) 3.0 mg $nTiO_2$/L and NOM, (f) 0.0 mg $nTiO_2$/L and (g) 0.0 mg $nTiO_2$/L and NOM. (e) represents the dose-response curve for an unaged Cu solution. The mean mortality for each treatment is denoted by an open circle, while the filled circle indicates the Cu EC_{50} value along with its 95% confidence interval

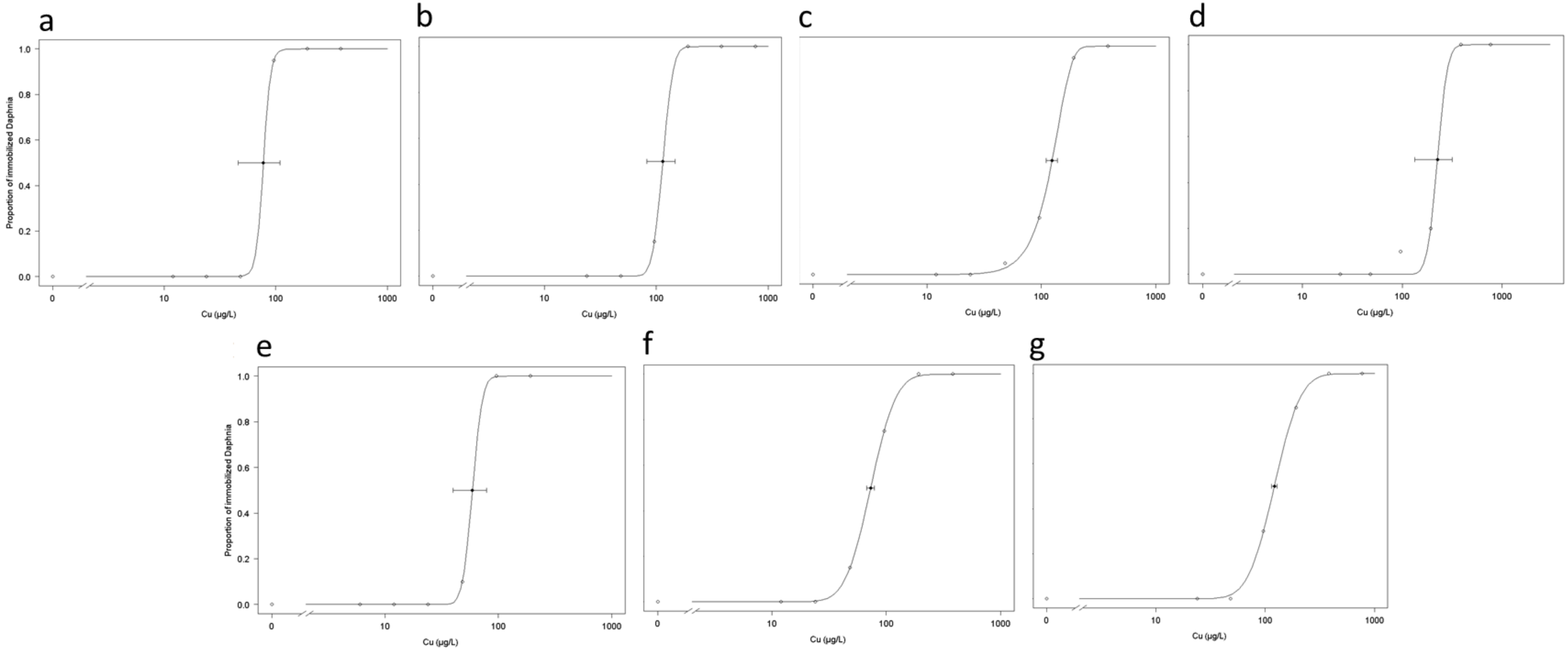

Fig. 3S Dose-Response curves based on nominal Cu concentrations for the 1 day aging (type 1) in combination with (a) 0.6 mg $nTiO_2$/L, (b) 0.6 mg $nTiO_2$/L and NOM, (c) 3.0 mg $nTiO_2$/L, (d) 3.0 mg $nTiO_2$/L and NOM, (f) 0.0 mg $nTiO_2$/L and (g) 0.0 mg $nTiO_2$/L and NOM. (e) represents the dose-response curve for an unaged Cu solution. The mean mortality for each treatment is denoted by an open circle, while the filled circle indicates the Cu EC_{50} value along with its 95% confidence interval

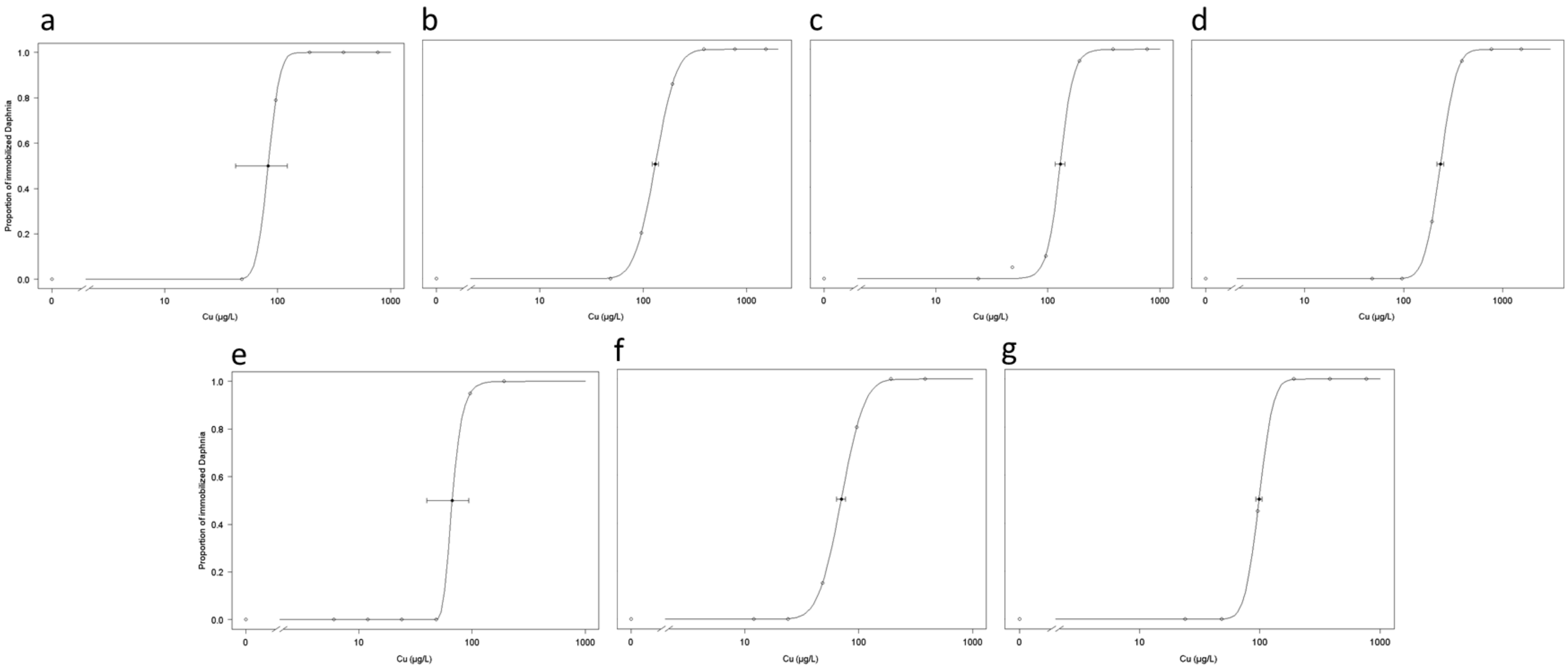

Fig. 4S Dose-Response curves based on nominal Cu concentrations for the 3 days aging (type 1) in combination with (a) 0.6 mg $nTiO_2$/L, (b) 0.6 mg $nTiO_2$/L and NOM, (c) 3.0 mg $nTiO_2$/L, (d) 3.0 mg $nTiO_2$/L and NOM, (f) 0.0 mg $nTiO_2$/L and (g) 0.0 mg $nTiO_2$/L and NOM. (e) represents the dose-response curve for an unaged Cu solution. The mean mortality for each treatment is denoted by an open circle, while the filled circle indicates the Cu EC_{50} value along with its 95% confidence interval

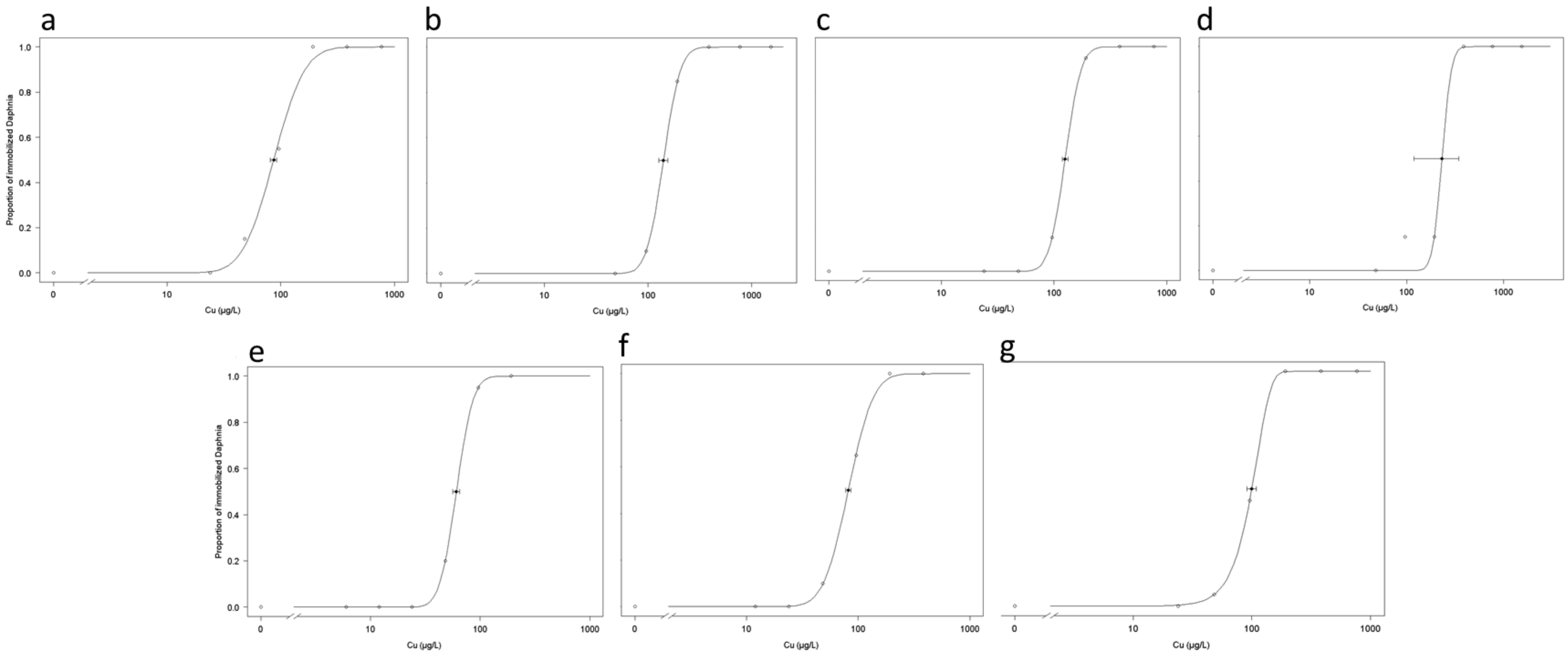

Fig. 5S Dose-Response curves based on nominal Cu concentrations for the 6 days aging (type 1) in combination with (a) 0.6 mg $nTiO_2$/L, (b) 0.6 mg $nTiO_2$/L and NOM, (c) 3.0 mg $nTiO_2$/L, (d) 3.0 mg $nTiO_2$/L and NOM, (f) 0.0 mg $nTiO_2$/L and (g) 0.0 mg $nTiO_2$/L and NOM. (e) represents the dose-response curve for an unaged Cu solution. The mean mortality for each treatment is denoted by an open circle, while the filled circle indicates the Cu EC_{50} value along with its 95% confidence interval

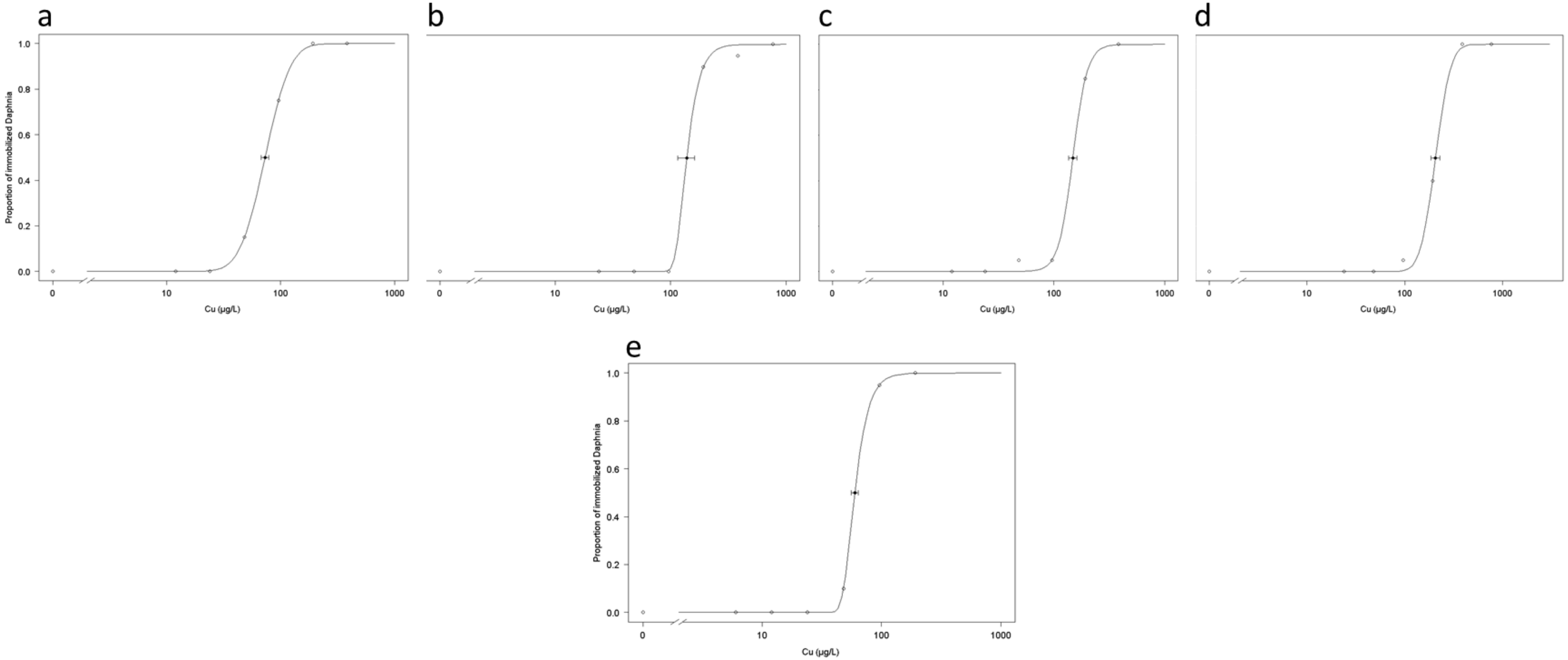

Fig. 6S Dose-Response curves based on nominal Cu concentrations for the 1 day aging (type 2) in combination with (a) 0.6 mg $nTiO_2$/L, (b) 0.6 mg $nTiO_2$/L and NOM, (c) 3.0 mg $nTiO_2$/L, (d) 3.0 mg $nTiO_2$/L and NOM. (e) represents the dose-response curve for an unaged Cu solution. The mean mortality for each treatment is denoted by an open circle, while the filled circle indicates the Cu EC_{50} value along with its 95% confidence interval

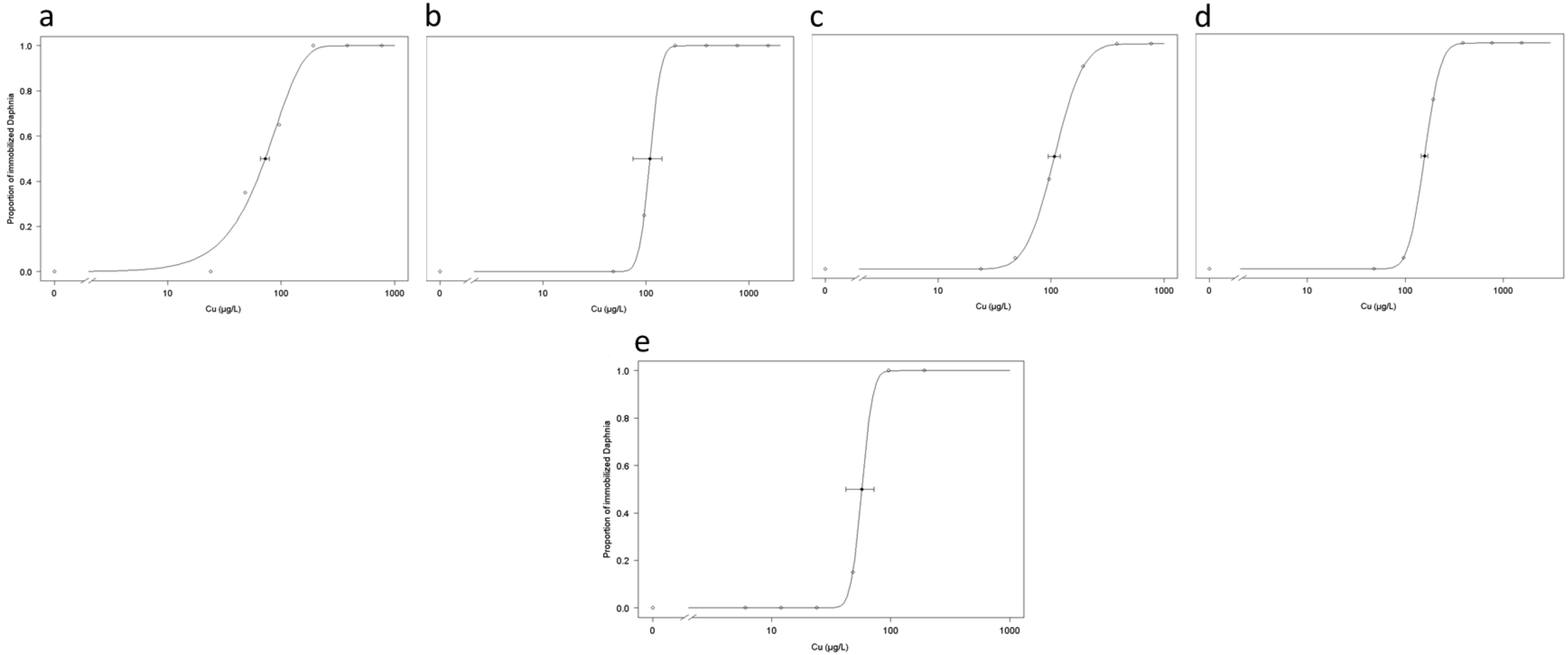

Fig. 7S Dose-Response curves based on nominal Cu concentrations for the 3 days aging (type 2) in combination with (a) 0.6 mg nTiO_2/L, (b) 0.6 mg nTiO_2/L and NOM, (c) 3.0 mg nTiO_2/L, (d) 3.0 mg nTiO_2/L and NOM. (e) represents the dose-response curve for an unaged Cu solution. The mean mortality for each treatment is denoted by an open circle, while the filled circle indicates the Cu EC_{50} value along with its 95% confidence interval

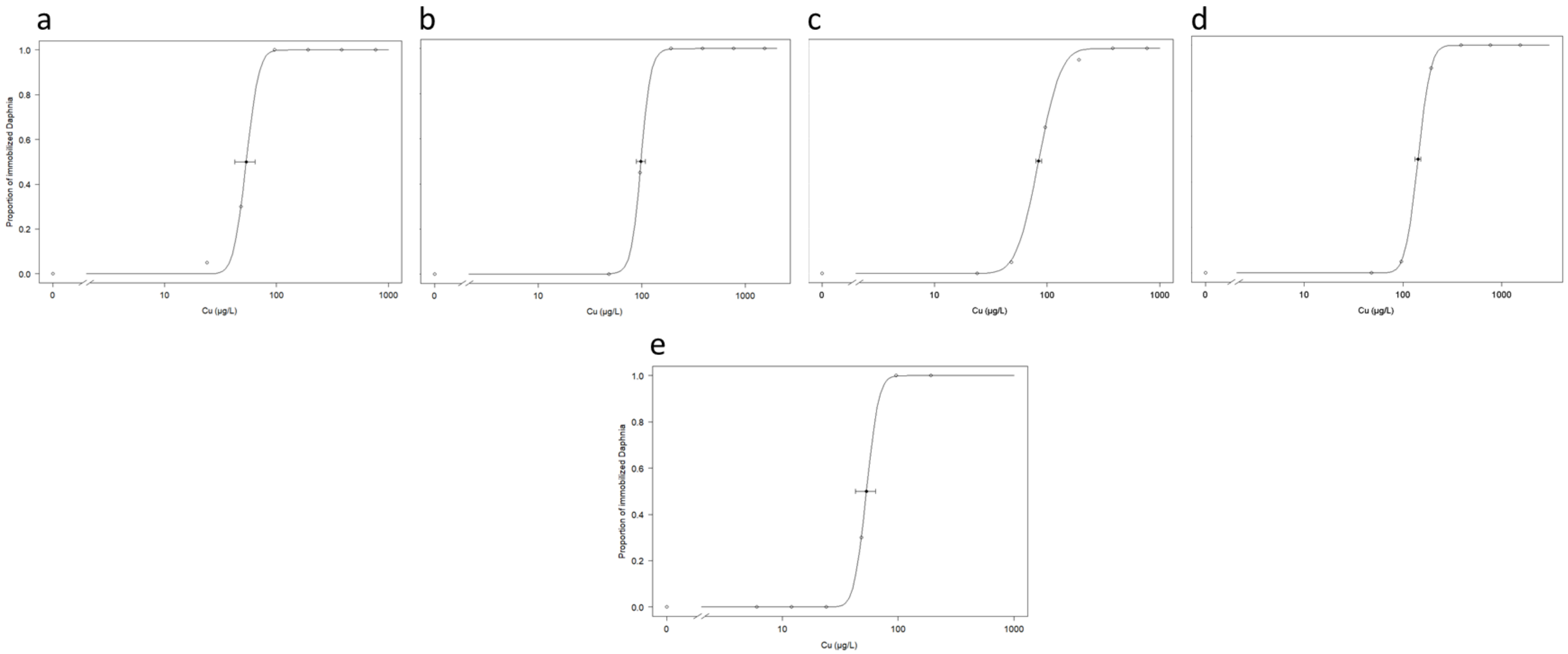

Fig. 8S Dose-Response curves based on nominal Cu concentrations for the 6 days aging (type 2) in combination with (a) 0.6 mg $nTiO_2$/L, (b) 0.6 mg $nTiO_2$/L and NOM, (c) 3.0 mg $nTiO_2$/L, (d) 3.0 mg $nTiO_2$/L and NOM. (e) represents the dose-response curve for an unaged Cu solution. The mean mortality for each treatment is denoted by an open circle, while the filled circle indicates the Cu EC_{50} value along with its 95% confidence interval

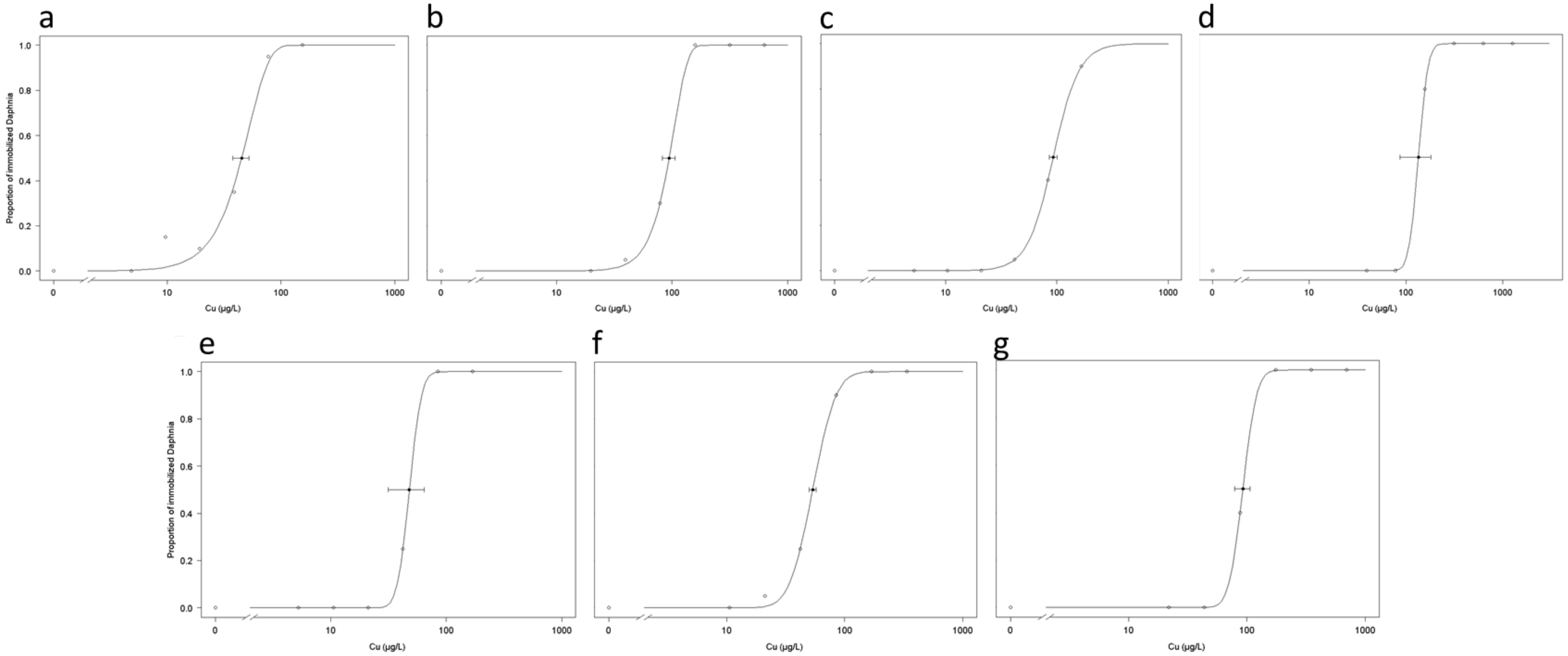

Fig. 9S Dose-Response curves considering the measured Cu concentrations via ICP-OES for the 0 day aging in combination with (a) 0.6 mg $nTiO_2$/L, (b) 0.6 mg $nTiO_2$/L and NOM, (c) 3.0 mg $nTiO_2$/L, (d) 3.0 mg $nTiO_2$/L and NOM, (f) 0.0 mg $nTiO_2$/L and (g) 0.0 mg $nTiO_2$/L and NOM. (e) represents the dose-response curve for an unaged Cu solution. The mean mortality for each treatment is denoted by an open circle, while the filled circle indicates the normalized Cu EC_{50} value along with its 95% confidence interval

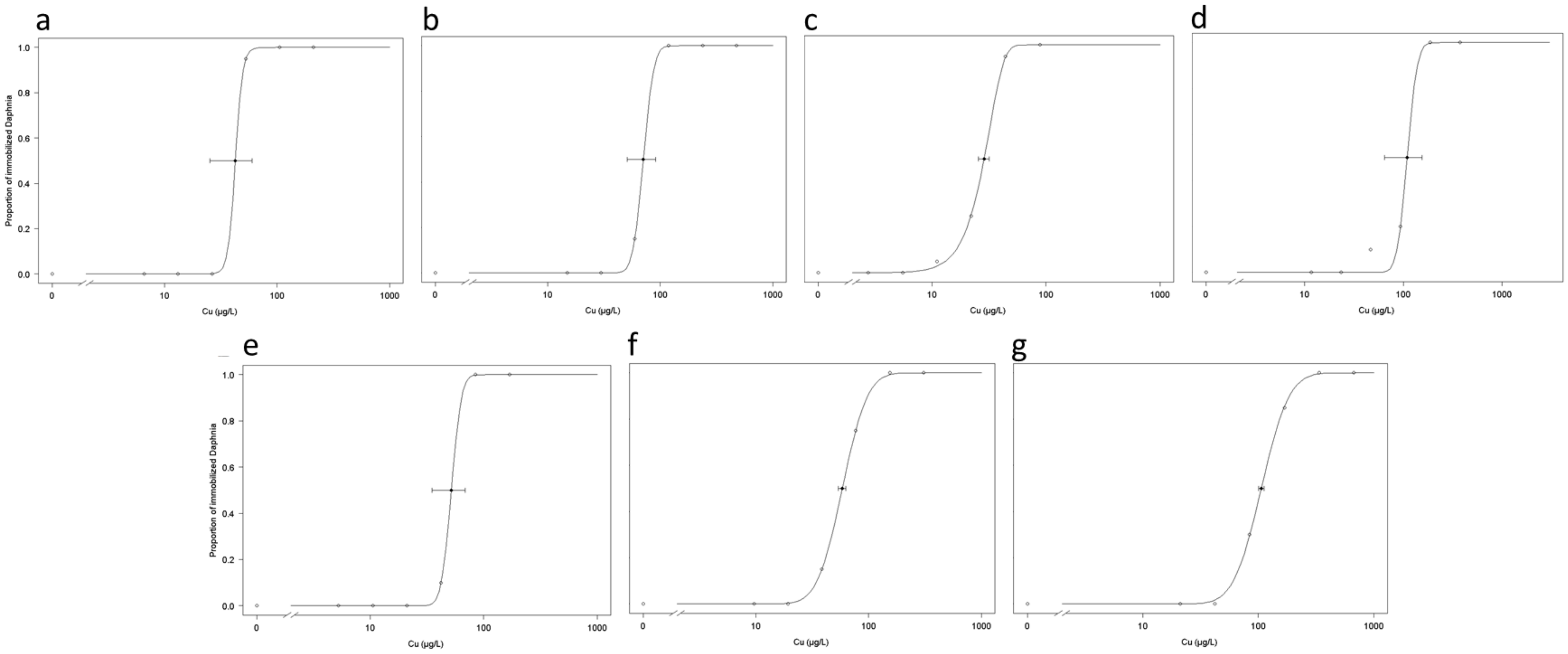

Fig. 10S Dose-Response curves considering the measured Cu concentrations via ICP-OES for the 1 day aging (type 1) in combination with (a) 0.6 mg $nTiO_2$/L, (b) 0.6 mg $nTiO_2$/L and NOM, (c) 3.0 mg $nTiO_2$/L, (d) 3.0 mg $nTiO_2$/L and NOM, (f) 0.0 mg $nTiO_2$/L and (g) 0.0 mg $nTiO_2$/L and NOM. (e) represents the dose-response curve for an unaged Cu solution. The mean mortality for each treatment is denoted by an open circle, while the filled circle indicates the normalized Cu EC_{50} value along with its 95% confidence interval

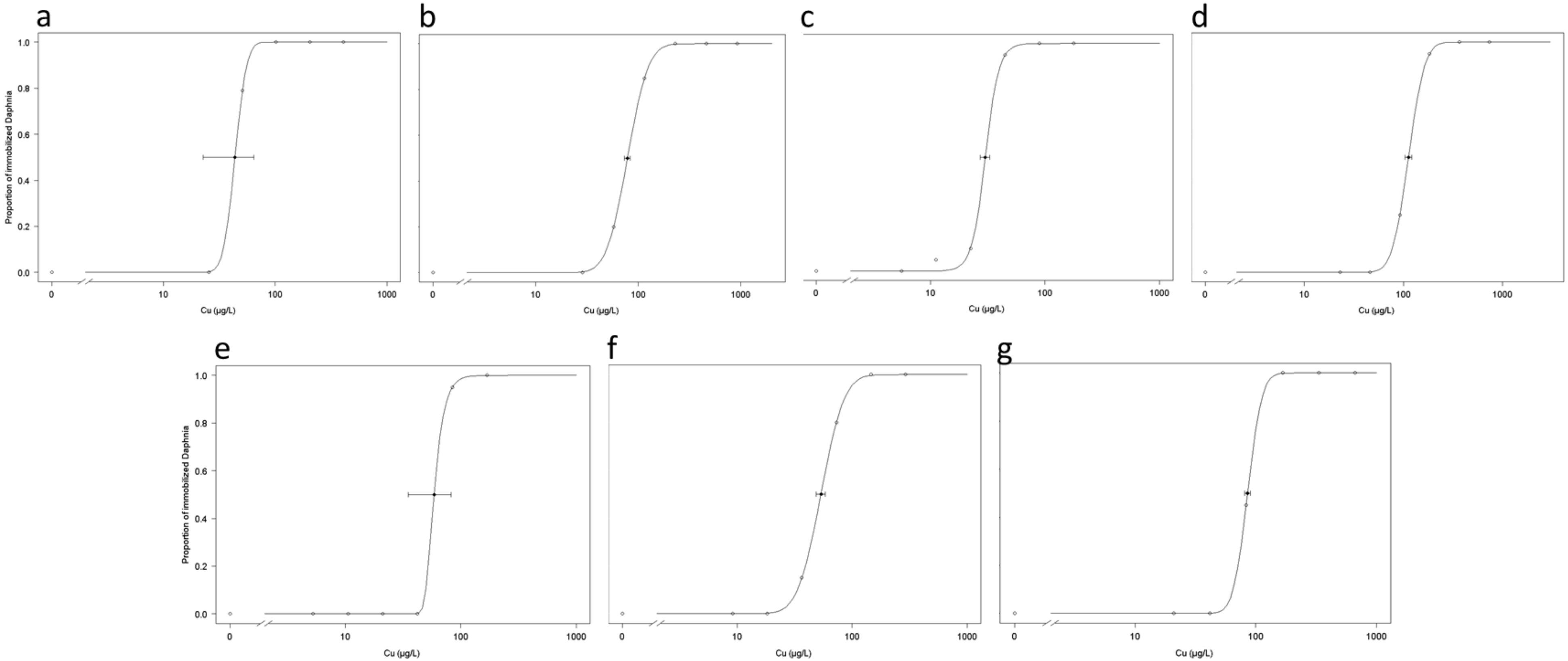

Fig. 11S Dose-Response curves considering the measured Cu concentrations via ICP-OES for the 3 days aging (type 1) in combination with (a) 0.6 mg $nTiO_2$/L, (b) 0.6 mg $nTiO_2$/L and NOM, (c) 3.0 mg $nTiO_2$/L, (d) 3.0 mg $nTiO_2$/L and NOM, (f) 0.0 mg $nTiO_2$/L and (g) 0.0 mg $nTiO_2$/L and NOM. (e) represents the dose-response curve for an unaged Cu solution. The mean mortality for each treatment is denoted by an open circle, while the filled circle indicates the normalized Cu EC_{50} value along with its 95% confidence interval

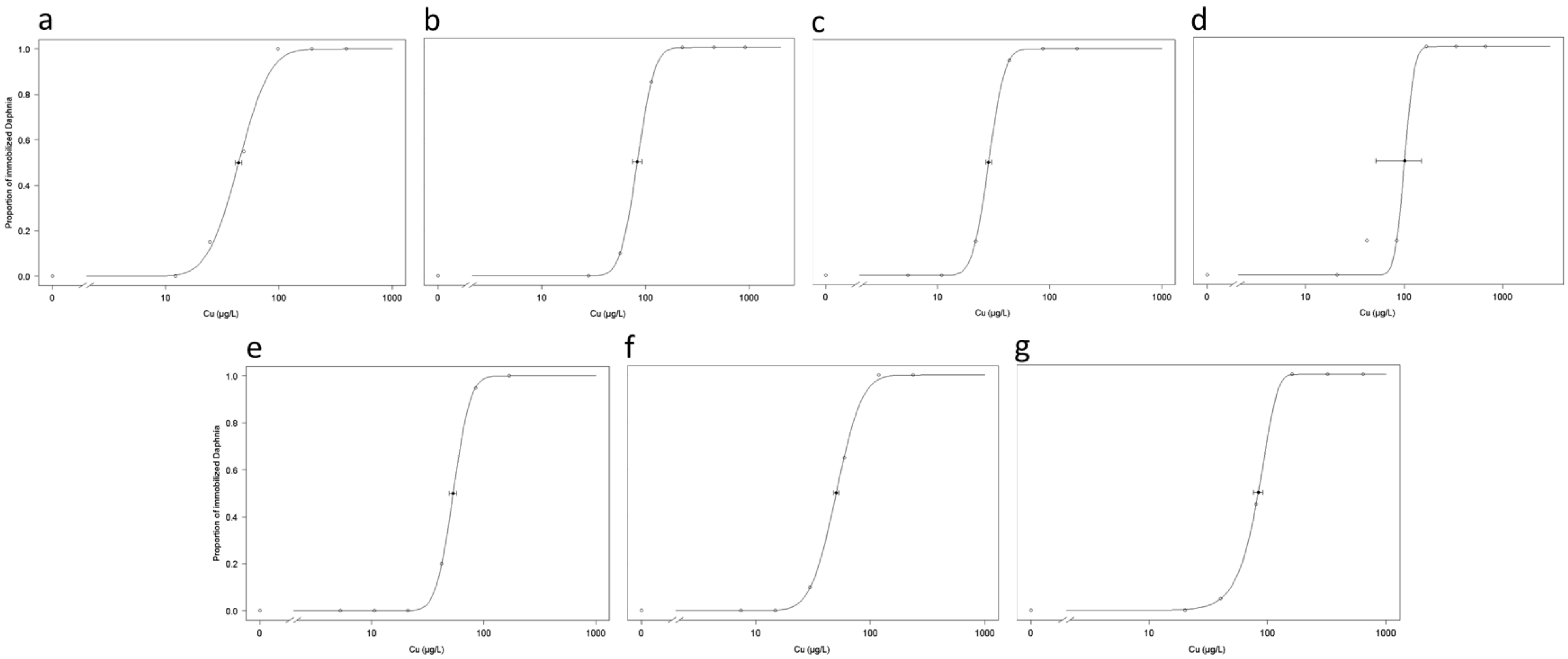

Fig. 12S Dose-Response curves considering the measured Cu concentrations via ICP-OES for the 6 days aging (type 1) in combination with (a) 0.6 mg $nTiO_2$/L, (b) 0.6 mg $nTiO_2$/L and NOM, (c) 3.0 mg $nTiO_2$/L, (d) 3.0 mg $nTiO_2$/L and NOM, (f) 0.0 mg $nTiO_2$/L and (g) 0.0 mg $nTiO_2$/L and NOM. (e) represents the dose-response curve for an unaged Cu solution. The mean mortality for each treatment is denoted by an open circle, while the filled circle indicates the normalized Cu EC_{50} value along with its 95% confidence interval

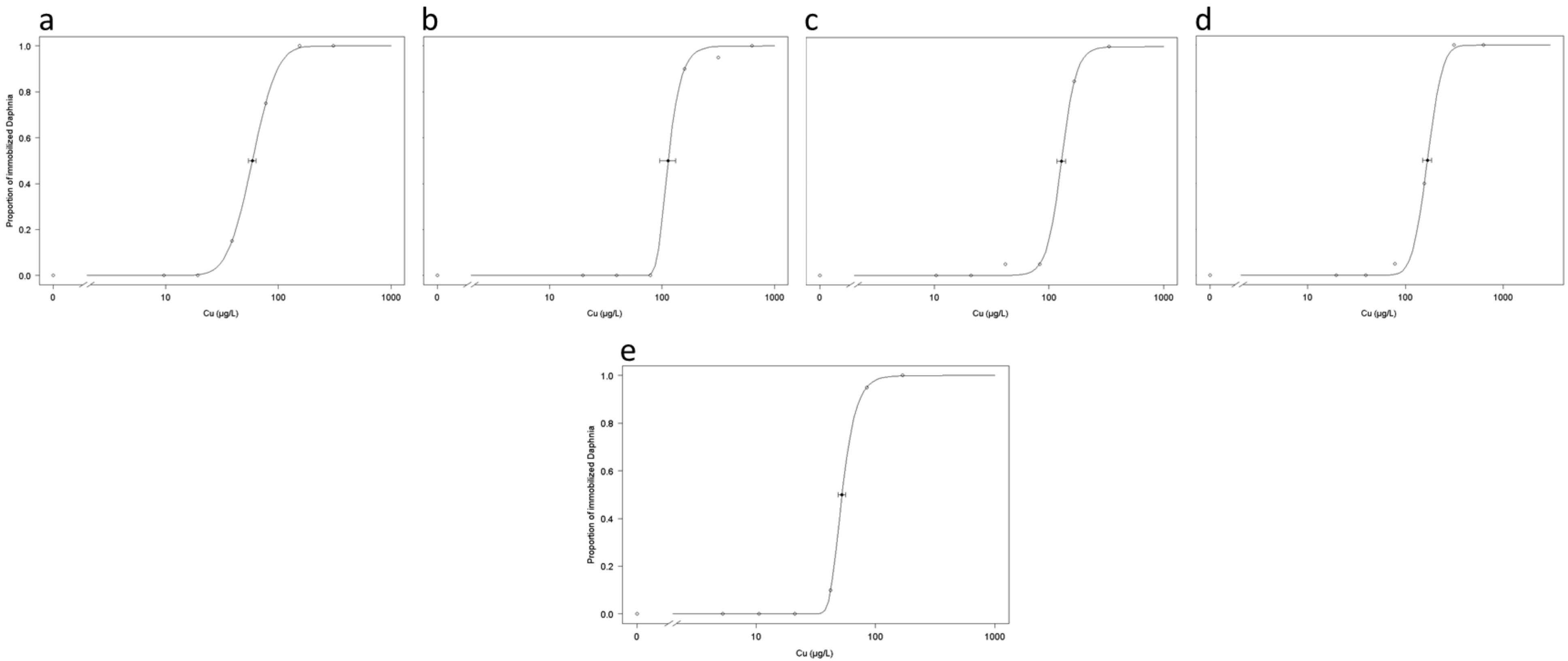

Fig. 13S Dose-Response curves considering the measured Cu concentrations via ICP-OES for the 1 day aging (type 2) in combination with (a) 0.6 mg $nTiO_2$/L, (b) 0.6 mg $nTiO_2$/L and NOM, (c) 3.0 mg $nTiO_2$/L, (d) 3.0 mg $nTiO_2$/L and NOM. (e) represents the dose-response curve for an unaged Cu solution. The mean mortality for each treatment is denoted by an open circle, while the filled circle indicates the normalized Cu EC_{50} value along with its 95% confidence interval

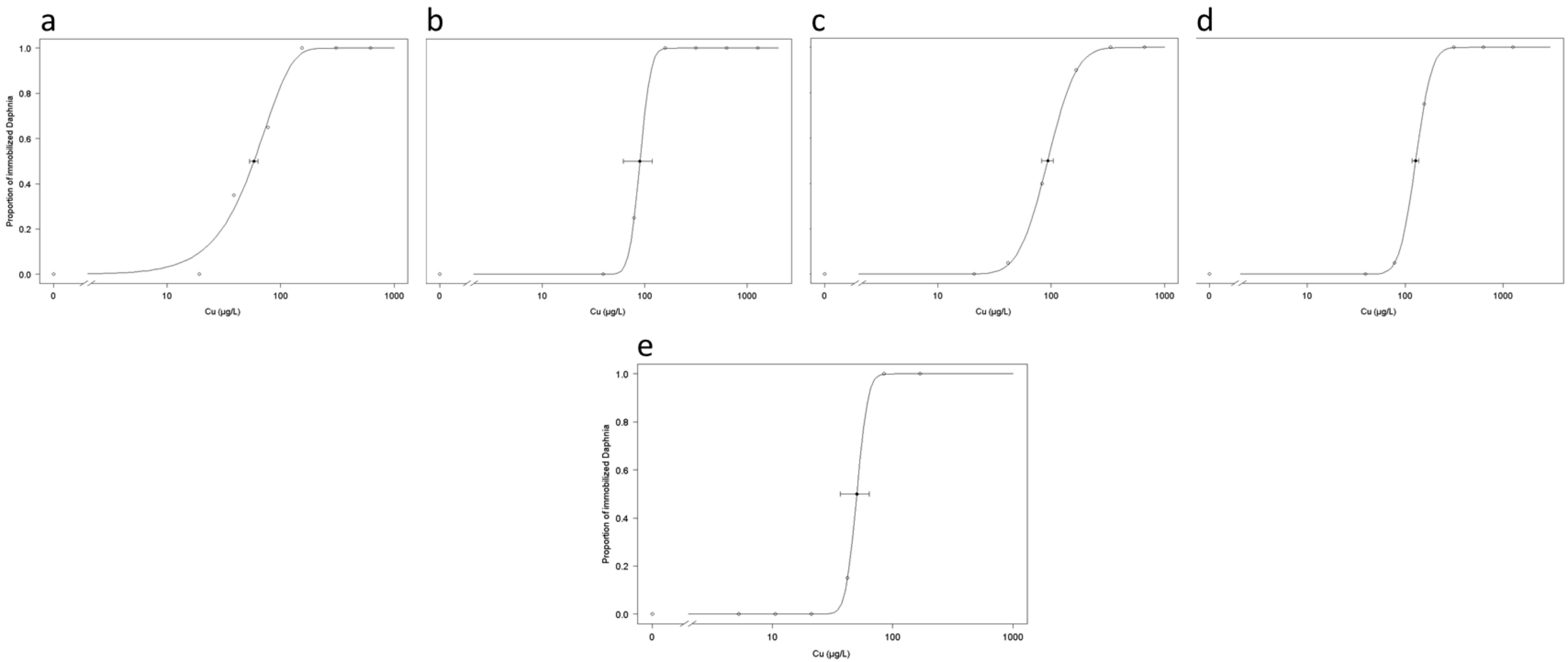

Fig. 14S Dose-Response curves considering the measured Cu concentrations via ICP-OES for the 3 days aging (type 2) in combination with (a) 0.6 mg $nTiO_2$/L, (b) 0.6 mg $nTiO_2$/L and NOM, (c) 3.0 mg $nTiO_2$/L, (d) 3.0 mg $nTiO_2$/L and NOM. (e) represents the dose-response curve for an unaged Cu solution. The mean mortality for each treatment is denoted by an open circle, while the filled circle indicates the normalized Cu EC_{50} value along with its 95% confidence interval

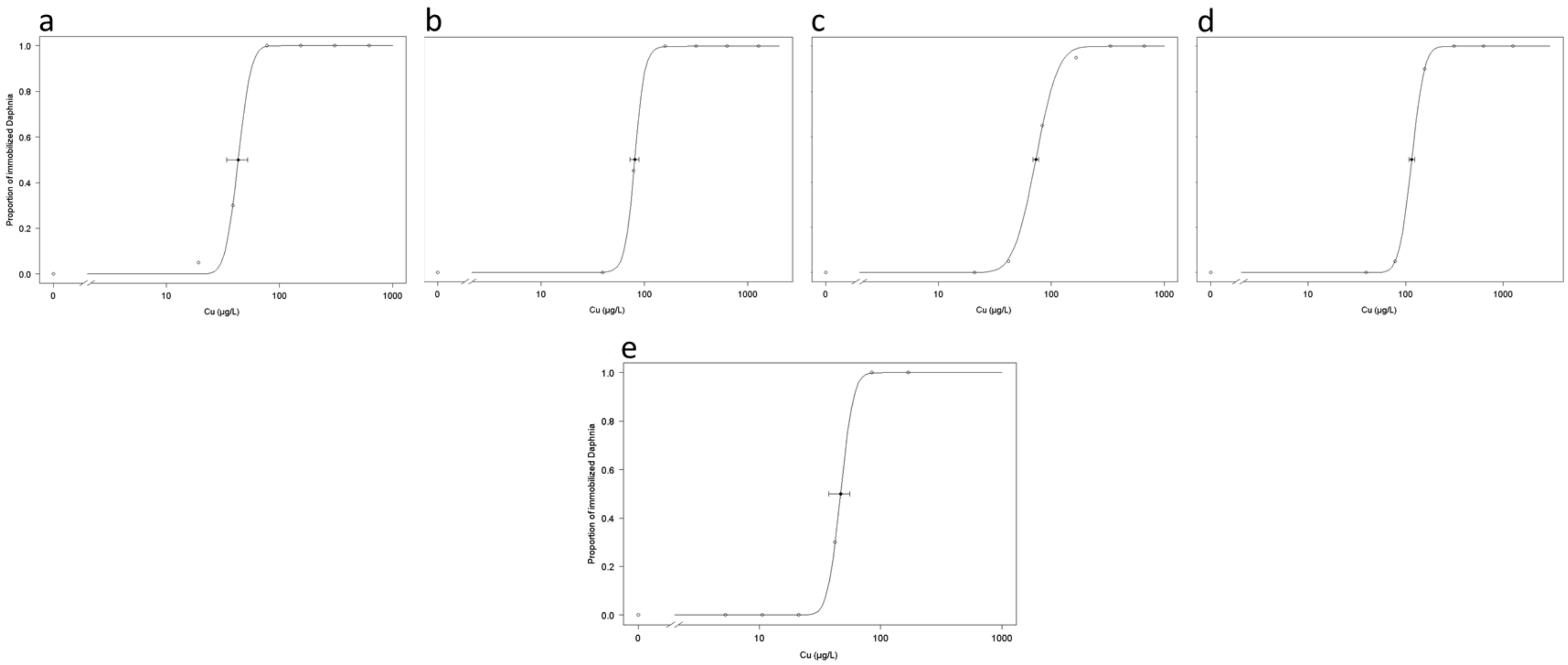

Fig. 15S Dose-Response curves considering the measured Cu concentrations via ICP-OES for the 6 days aging (type 2) in combination with (a) 0.6 mg $nTiO_2$/L, (b) 0.6 mg $nTiO_2$/L and NOM, (c) 3.0 mg $nTiO_2$/L, (d) 3.0 mg $nTiO_2$/L and NOM. (e) represents the dose-response curve for an unaged Cu solution. The mean mortality for each treatment is denoted by an open circle, while the filled circle indicates the normalized Cu EC_{50} value along with its 95% confidence interval

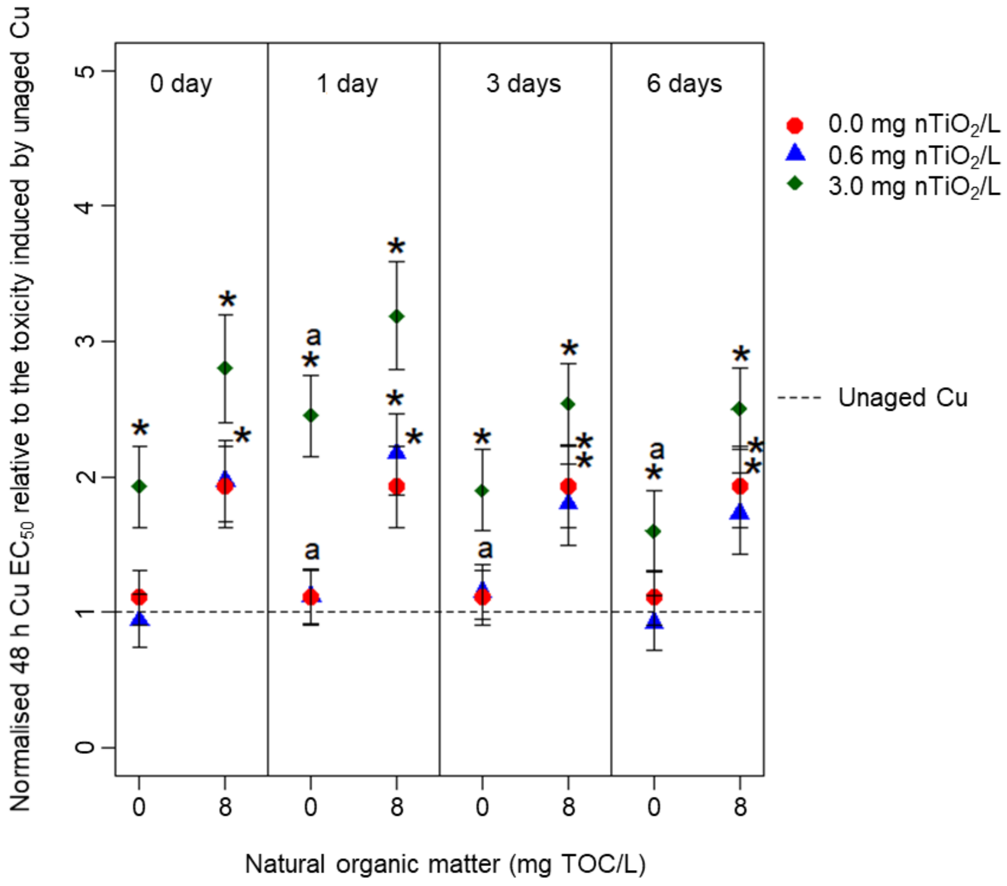

Fig. 16S Changes in the Cu 48 h EC_{50} of *D. magna* normalised to the toxicity induced by the unaged Cu solution (the reference absolute 48 h EC_{50} range 46.7 ± 4.5 to 52.4 ± 1.8 µg/L) and reflect the impact of $nTiO_2$ (0.0, 0.6 or 3.0 mg/L), NOM (0 or 8 mg/L) and aging duration (0, 1, 3, 6 days) for the type 2 aging scenario. The EC_{50} values are based on measured Cu concentrations. Asterisk indicates a significant difference of the respective EC_{50} value relative to the bioassays testing for the effects of unaged Cu. The 'a' indicates a significant difference of the respective EC_{50} values relative to the same combinations of treatments but aged for 0 days

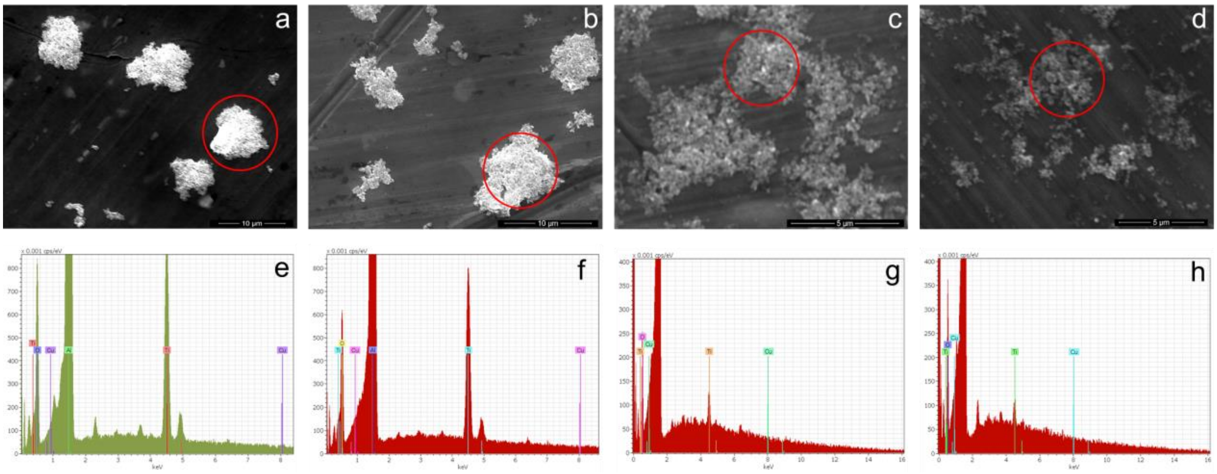

Fig. 17S Scanning electron microscopic (SEM) images of 1 day aged $nTiO_2$ (3.0 mg/L) agglomerates (a) in the presence of Cu (96 µg/L), (b) in the absence of Cu, (c) in the presence of NOM and Cu (192 µg/L), and (d) in the presence of NOM but absence of Cu along with respective energy dispersive X-ray (EDX) analysis data (e), (f), (g) and (h). The lack of Cu peaks in (e) and (g) are likely driven by the relatively low amount of Cu applied relative to the concentration of $nTiO_2$. Consequently, any Cu potentially adsorbed to the NP surface was below the detection limit (which has not been determined). Specification of the instrument: Quanta 250 scanning electron microscope (FEI Company, Eindhoven) at a 1200-fold magnification

Reference

Seitz F, Lüderwald S, Rosenfeldt RR, Schulz R, Bundschuh M (2015) Aging of TiO_2 nanoparticles transiently increases their toxicity to the pelagic microcrustacean *Daphnia magna*. PLoS One 10:e0126021. https://doi.org/10.1371/journal.pone.0126021
